# Chemical compositions of *Eucalyptus* sp. Essential oils and the evaluation of their combinations as a promising treatment against ear bacterial infections

**DOI:** 10.1186/s12906-024-04494-2

**Published:** 2024-06-07

**Authors:** Elaissi Ameur, Moumni Sarra, Derbali Yosra, Khouja Mariem, Abid Nabil, Jlasssi Ibrahim, Khaloud Mohammed Alarjani, Frederic Lynen, Khouja Mohamed Larbi

**Affiliations:** 1 Chemical, Pharmacological and Gallenic Development Laboratory, Faculty of Pharmacy, University of Monastir, Avenue Avicennne, 5019 Monastir, Tunisia; 2 Water, and Forestry. INRGREF. Laboratory of Management and Valorization of Forest Resources, University of Carthage, The National Research Institute of Rural Engineering, 2080 Ariana, Tunisia; 3 Laboratory of Transmissible Diseases and Biological Active Substances LR99ES27, Faculty of Pharmacy, University of Monastir, Monastir, Tunisia; 4 Department of Botany and Microbiology, College of Science, King Saud University, 11451 Riyadh, Saudi Arabia; 5 Separation Science Group, Department of Organic and Macromolecular Chemistry, Faculty of Sciences, Ghent University, Krijgslaan 281-S4 Bis, B-9000 Ghent, Belgium

**Keywords:** *Eucalyptus* essential oils, EOs blends, Antibacterial activity, Ear infection, Principal Component Analysis (PCA), Hierarchical Cluster Analysis (HCA)

## Abstract

**Background:**

The chemical composition and biological activities of *Eucalyptus* essential oils (EOs) have been documented in numerous studies against multiple infectious diseases. The antibacterial activity of individual *Eucalyptus* EOs against strains that cause ear infections was investigated in our previous study. The study's antibacterial activity was promising, which prompted us to explore this activity further with EO blends.

**Methods:**

We tested 15 combinations (9 binary combinations and 6 combinations of binary combinations) of *Eucalyptus* EOs extracted by hydrodistillation from eight Tunisian *Eucalyptus* species dried leaves against six bacterial strains responsible for ear infections: three bacterial isolates (*Haemophilus influenzae*, *Haemophilus parainfluenzae*, and *Klebsiella pneumoniae*) and three reference bacteria strains (*Pseudomonas aeruginosa*, ATTC 9027; *Staphylococcus aureus*, ATCC 6538; and *Escherichia coli*, ATCC 8739). The EOs were analyzed using GC/FID and GC/MS. The major compounds, as well as all values obtained from the bacterial growth inhibition assay, were utilized for statistical analysis.

**Results:**

The antibacterial activity of the EO blends exhibited significant variation within *Eucalyptus* species, bacterial strains, and the applied methods. Principal component analysis (PCA) and hierarchical cluster analysis (HCA), based on the diameters of the inhibition zone, facilitated the identification of two major groups and ten subgroups based on the level of antibacterial activity. The highest antibacterial activity was observed for the mixture of EOs extracted from *E. panctata*, *E. accedens*, and *E. cladoclayx* (paac) as well as *E. panctata*, *E. wandoo*, *E. accedens*, and *E. cladoclayx* (pwac) using the disc diffusion method. Additionally, significant activity was noted with EOs extracted from *E. panctata*, *E. wandoo* (pw) and *E. panctata*, *E. accedens* (pa) using the broth microdilution method.

**Conclusion:**

Our findings suggest that certain EO combinations (paac, pwac, pw, and pa) could be considered as potential alternative treatment for ear infections due to their demonstrated highly promising antibacterial activities.

**Supplementary Information:**

The online version contains supplementary material available at 10.1186/s12906-024-04494-2.

## Background

Ear infections stand out as the most prevalent ailment across all age groups, predominantly impacting both adults and young children [1]. Common bacterial strains associated with ear infections include *Streptococcus pneumoniae*, *Staphylococcus aureus*, *Hemophilus influenzae*, and *Moraxella catarrhalis*. Acute otitis media is typically linked to these strains, while chronic otitis media is more frequently associated with *Pseudomonas aeruginosa*, *S. aureus*, *Proteus mirabilis*, *Klebsiella pneumoniae*, and *Escherichia coli*. These bacteria not only contribute to the development of acute conditions but also lead to complications such as ear discharges, ruptured eardrums, and other issues. Additionally, they induce inflammation and pain in the middle ear [2].

Ear infections exert a considerable negative impact on health, serving as a significant contributor to avoidable hearing loss and representing a prevalent clinical issue [3]. Globally, millions of children grapple with debilitating hearing loss, estimated by the World Health Organization (WHO) to affect over 5% of the population [3]. A noteworthy aspect is that many instances of this condition are either curable or preventable. However, the escalating resistance to antibiotics displayed by these bacterial strains poses a growing challenge in successfully treating ear infections. This underscores the urgent need for research in this field to counteract resistance and safeguard public health.

Plant essential oils (EOs) are natural mixtures of abundant components such as terpenes, terpenoids, aromatic, and aliphatic constituents. The use of EOs as alternative for drugs was reported in many infection cases. Thyme oil were found to be the most effective agents against fungal isolates and they constitute a promising tool for the management of fungal infection causing the otitis media [4]. According to a small study, drops crafted from a blend of essential oils extracted from cloves, English lavender, and herb-Robert demonstrated comparable effectiveness to an antibiotic ear drop, commonly prescribed for treating ear infections [5]. 

Nonetheless, some bacterial strains showed demonstrated heightened resistance to EOs following repeated treatment [6]. Consequently, EO combinations exert a multifarious impact on microbial cells, rendering them potent antimicrobials. This characteristic not only expands the repertoire of potential natural antimicrobial agents but also offers an alternative to antibiotics, enhancing efficacy without fostering the development of resistance [7, 8].

While some researchers have investigated the outcomes of blending *Eucalyptus* EOs with oils from different genera, comprehensive studies on combining different EOs from *Eucalyptus* are lacking. Notably, previous research has demonstrated synergistic activity against E. coli and S. aureus in binary mixes of E. globulus EO with those of Cinnamomum verum and Glycyrrhiza glabra [9]. Additionally, diverse binary combinations of EOs from different *Eucalyptus* species yielded varying results against *Paenibacillus amylolyticus* and *Bacillus cereus*, including synergistic effects with *Thymus vulgaris* and *Cinnamomum cassia* Siebold, additive effects with *Citrus reticulata* (Blanco) M. Hiroe and *E. globulus*, and indifferent effects with *Cinnamomum cassia* Siebold and *E. globulus* [10].

In our earlier study, we we investigated the impact of eight different Eucalyptus EOs on bacterial strains associated with otitis [11]. Notably, extracts from *E. melliodora*, *E. bosistoana*, and *E. robusta* exhibited potential antibacterial activity against both *K. pneumoniae* and *E. coli*.

Therefore, the primary objective of this study was to evaluate the antimicrobial efficacy of Eucalyptus EOs against specific bacterial strains implicated in otitis infections, considering various combinations. The central focus of the research lies in understanding the relationship between the chemical composition of these EOs and the antibacterial activity of both their binary combinations and combinations of binary combinations.

## Methods

### Plant material

Eight species of Eucalyptus L'Hér, which were collected in June 2017 from Tunisia, were used in our study. The clean and mature leaves were collected from the following species: E. accedens Fitzg, E. robusta Sm., E. punctata DC., E. melliodora, E. lesouefii Maiden, E. cladocalyx F. Muell, E. bosistoana F. Muell., and E. wandoo. Botanical voucher specimens were identified by Professor Mohamed Khouja Larbi and stored in the Pharmacognosy laboratory of the Faculty of Pharmacy in Monastir (Tunisia) under the following reference numbers: 0173, 0174, 0175, 0176, 0177, 0178, 0179, 180.

### Extraction of essential oils

The EOs were extracted using an approved standard equipment specified by the European Pharmacopoeia. This involved hydrodistilling 100 g of coarsely crushed leaves for 4 h. Hydrodistillation was conducted in triplicate for each sample. The extracted EO was collected, dried with Na2SO4, and subsequently stored at 4 °C for analysis.

### Gas chromatography (GC) and Gas chromatography-mass spectrometry (GC–MS) Analyses

The EO extracts were subsequently analyzed using GC and GC–MS in triplicate. The GC analysis was performed on a Hewlett-Packard 6890 apparatus equipped with an FID and an apolar HP5 capillary column. The experimental parameters were as follows: the oven temperature was programmed to start at 60 °C for 1 min, then gradually increased from 60 °C to 250 °C at a rate of 3 °C/min, and held isothermally at 250 °C for 3 min. The injector temperature was set at 250 °C whereas the detector temperature was set at 280 °C, and the used carrier gas was N2 at a flow rate of 1.2 mL/min. For each sample, 1 μL (diluted to 10% EO in purified hexane) was injected for analysis. The relative concentration was calculated using HP ChemStation software, which assimilates the percentages of peak areas to the percentages of various constituents. Retention indices (RI) were determined according to the retention time (Rt) of a series of n-alkanes (C9-C28).

### Bacterial strains

The current study included three ATCC microorganisms [*P. aeruginosa* (ATTC 9027), *S. aureus* (ATCC 6538), and *E. coli* (ATCC 8739)] and three clinical bacterial isolates (*H. influenzae*, *H. parainfluenzae*, and *K. pneumoniae*). The ATCC strains were available in the culture collection of the Laboratory of Transmissible Diseases and Biologically Active Substances (Faculty of Pharmacy, Monastir, Tunisia), while the clinical strains were kindly provided by the Microbiology and Immunology Laboratory (EPS Farhat Hachad, Sousse, Tunisia).

### Antibacterial assays of EOs mixtures

#### Disc diffusion method

The Disc agar diffusion technique was used to assess the antibacterial activities of several EOs using bacterial inoculums of 0.5 McFarland; Mueller Hinton (MH) supplemented with 5% sheep blood. *P. aeruginosa*, *E. coli*, and *S. aureus*, however, did not undergo enrichment. The 90 mm inoculation plates were covered with absorbent discs (Whatman disc n°3, 6 mm diameter) that had been impregnated with 10 μL of each EO.. Every essay had a positive control disc containing 10 μg of Gentamicine®. Upon incubation for 24 h at 37 °C, the inhibitory zones were measured and shown in millimeters. The findings were interpreted using the previously mentioned parameters [12, 13]: diameter ≤ 8 mm indicated not sensitive or no inhibitory effect (-); diameter between 8 and 14 mm indicated sensitive ( +) or mild inhibitory effect; diameter between 14 and 20 mm indicated very sensitive or moderate inhibitory effect (+ +); diameter ≥ 20 mm indicated extremely sensitive or strong inhibitory effect (+ + +). Every experiment was conducted in triplicate, and the mean ± standard errors of mean was used to express the results.

#### Broth microdilution method

The Broth microdilution method was carried out to calculate the Minimum Inhibitory Concentration (MIC) and the Minimum Bactericidal Concentration (MBC) of the tested *Eucalyptus* EOs according to the National Committee for Clinical Laboratory Standard [14].

An overnight culture (37 °C) of each tested strain was prepared by adjusting the turbidity of each bacterial culture to reach an optical density of 0.5 McFarland standards. 100 μL from each EO diluted in DMSO (10%), initially prepared at a concentration of 931 mg/mL, were added into the third well, followed by two-fold serial dilutions in MH Broth medium until the 12th well. Subsequently, 80 μL of MH, 10 μL of the inoculum, and 10 μL of 0.02% resazurin solution were added into each well. The skipped first and the second well were reserved for negative and positive controls, respectively. Negative control well contained bacteria only in the MH broth medium whereas, positive control well contained 10 μg/ mL of Gentamicin® antibiotics.

After incubation for 24 h at 37 °C, the bacterial growth was characterized by color change from blue to pink. The MIC was defined as the lowest concentration that completely inhibits visible cell growth during 24 h incubation at 37 °C (blue colored well). To determine the minimum bactericidal concentration (MBC) values, 10 μL of each culture medium with no visible growth were removed and inoculated in MH plates. After incubation for 18–24 h at 37 °C, the number of surviving organisms was determined. The MBC was defined as the lowest concentration at which 99.9% of the bacteria culture were killed [15, 16]. As for other analyses, the experiments were performed in triplicate. The tested EOs are considered bactericidal if the ratio MBC / MIC < 4 and bacteriostatic if the ration MBC / MIC > 4.

### Effect of the EOs blends on their antibacterial activities

#### Combination's criteria

The two used methods (disc diffusion and broth microdilution) were carried out using two types of combinations:

- *Binary combinations*: The antibacterial effect of EO mixtures (1:1, v/v) was evaluated based upon their previously calculated inhibition zone diameter (izd) and according to the following choices: i) mixing previous none active EOs; ii) mixing EOs with no activity with those showing high activity; iii) mixing EOs with the most significant activity.
- *Combinations of binary combinations*: We used only the binary combinations that showed a izd value ≥ 15.00 mm; however, due to the small available quantity of the remaining EOs, we limit our study to 6 combinations of binary combinations.

### Evaluation of antibacterial effect

#### Disc diffusion method

The antibacterial effect of EO mixtures (1:1, v/v) was evaluated based on their antibacterial activity expressed by their inhibition zone diameter (izd).

According to the izd values of EO mixtures, three effects could be observed [17]: Synergistic effect: when the effect of (A + B) > effect A + effect B; Additive effect: when the effect of (A + B) = effect A + effect B; Antagonist effect: when the effect of (A + B) < effect A + effect B; where A and B are two EO samples.

We performed 15 combinations (9 binary and 6 combinations of binary combinations) against 6 bacterial strains (total = 90 effects). Thus, the percentage of each type of antibacterial effect (synergic, additive, or antagonism) is calculated according to the following formula:

$$\%\mathrm T\mathrm y\mathrm p\mathrm e\_\text{Effect}=\mathrm N\mathrm u\mathrm m\mathrm b\mathrm e\mathrm r\,\mathrm o\mathrm f\,\mathrm e\mathrm f\mathrm f\mathrm e\mathrm c\mathrm t\,\mathrm t\mathrm y\mathrm p\mathrm e/\mathrm T\mathrm o\mathrm t\mathrm a\mathrm l\,\mathrm n\mathrm u\mathrm m\mathrm b\mathrm e\mathrm r\,\mathrm o\mathrm f\,\mathrm e\mathrm f\mathrm f\mathrm e\mathrm c\mathrm t\mathrm s\ast100$$

### Broth microdilution method

The evaluation of the antibacterial effect is based on the fractional concentration indices (FICI), which allowed us to identify the type of interaction of the combined EOs against the tested bacterial strains. The FICI was calculated according to the following formula: [18]

FICI = FICA + FICB, where FICA = (MIC of the combination (A-B) / MICA alone) and FICB = (MIC of the combination (A-B)/MICB alone). The results were interpreted as a total synergistic effect (FICI ≤ 0.5), a partial synergism effect (0.5 < FICI ≤ 0.75), an additive effect (0.75 < FICI ≤ 1), an indifference (1 < FICI ≤ 4), and an antagonism effect (FICI > 4).

### Statistical analysis

The ANOVA test was used to compare the *Eucalyptus* species blends of EOs, the content of their chemical components, and the values of their izd against the tested bacterial strains. The level of statistical significance was determined at *p* < 0.05 using Duncan's multiple range tests. In order to identify the possibility of a relationship between the chemical composition of the EO mixtures and their antibacterial activities, all the values of the izd of bacteria growth inhibition were used for Principal Component Analysis (PCA) and Hierarchical Cluster Analysis (HCA) analyses using IBM SPSS Statistics for Windows, Version 23.0 (Armonk, NY: IBM Corp).

## Results

### Antibacterial activity of EOs mixtures

#### Disc diffusion method

The antibacterial activity of the *Eucalyptus* EO mixtures against six clinical and reference bacterial strains exhibited significant variation among the EOs (*p* ≤ 0.001). Post hoc Duncan's multiple range tests identified six overlapping groups (a-f) (Table 1). Assessment of the activities of EO mixtures, calculated in relation to the total number of observed effects, revealed that 12 combinations exhibited synergistic effects (13.33%), one combination (1.11%) had an additive effect, while the remaining combinations demonstrated antagonistic effects (85.55%).

**Table 1:** Effect of *Eucalyptus* essential oils combinations and the antibiotic (gentamicin) on ear infection bacterial growth

***Eucalyptus* species EOs**		***Bacterial strains and interpretation (Int)***											
	Gram-negative											Gram-positive	
**Individual EOs**	Ab*)	***Escherichia coli ATCC 8739***	**Int**	***Haemophilus influenzae***	**Int**	***Haemophilus parainfluenzae***	**Int**	***Klebsiella*** ***pneumoniae***	**Int**	***Pseudomonas aeruginosa ATCC 9027***	**Int**	***Staphylococcus aureus ATCC 6538***	**Int**
*E. accedens*	a*)	12,3 ± 3,8b*)		10,0 ± 1,0ab		12,7 ± 2,5b		7,0 ± 1,7a		6,3 ± 0,6a		9,3 ± 0,6a	
*E. bosistoana*	*b*	13,7 ± 1,5bc**)		6,0 ± 0,0a		7,3 ± 1,5ab		16,0 ± 1,7bcd		6,0 ± 0,0a		10,7 ± 4,0ab	
*E. cladocalyx*	*c*	15,7 ± 3,2bcd		6,0 ± 0,0a		6,3 ± 0,6a		15,0 ± 4,4bc		6,0 ± 0,0a		8,0 ± 2,6a	
*E. lesoufeii*	*l*	10,0 ± 2,0ab		9,3 ± 1,2ab		9,3 ± 2,1ab		8,7 ± 1,2a		8,3 ± 0,6a		13,3 ± 1,2b	
*E. melliodora*	*m*	19,7 ± 6,7de		6,3 ± 0,6a		7,0 ± 1,7ab		19,7 ± 2,9 cd		6,0 ± 0,0a		8,3 ± 2,1a	
*E. punctata*	*p*	6,0 ± 0,0a		11,7 ± 2,1b		10,7 ± 3,5ab		7,0 ± 0,0a		6,0 ± 0,0a		9,7 ± 3,2a	
*E. robusta*	*r*	20,7 ± 1,5de		10,0 ± 1,0 ab		10,0 ± 2,6ab		16,0 ± 4,6bcd		7,7 ± 1,5a		6,3 ± 0,6a	
*E. wandoo*	*w*	18,3 ± 0,6cde		9,0 ± 1,0ab		9,3 ± 0,6ab		12,0 ± 1,7ab		6,0 ± 0,0a		9,0 ± 1,0a	
**Binary EOs combinations**													
*E. bosistoana* + *E. punctata*	*bp*	10.0 ± 0.0ab	A***)	14,3 ± 3,2ab	A	8,0 ± 2,0a**)	A	9,0 ± 3,6ab	A	15,0 ± 0,0f	S	14,7 ± 0,6 cd	A
*E. accedens* + *E. cladocalyx*	ac	10.7 ± 1.2b	A	15,7 ± 2,1ab	A	8,3 ± 0,6a	A	8,0 ± 0,0ab	A	14,3 ± 0,6f	S	22,3 ± 2,5e	S
*E. wando* + *E. cladocalyx*	wc	6.0 ± 0.0a	A	10,3 ± 4,5a	A	8,0 ± 2,0a	A	6,0 ± 0,0a	A	10,0 ± 0,0bcd	A	8,7 ± 2,3ab	A
*E. bosistoana* + *E. lesouefii*	bl	8.7 ± 1.2ab	A	12,7 ± 3,1ab	A	15,7 ± 3,2bc	A	18,0 ± 2,0d	A	10,0 ± 0,0bcd	A	13,3 ± 2,3bcd	A
*E. bosistoana* + *E. melliodora*	bm	8.0 ± 0.0ab	A	11,3 ± 2,3a	A	15,3 ± 1,5bc	S	12,7 ± 1,2bc	A	13,7 ± 1,2ef	S	17,3 ± 0,6d	A
*E. punctata* + *E. melliodora*	pm	8.7 ± 1.2ab	A	9,3 ± 2,3a	A	16,0 ± 1,0bc	A	11,0 ± 3,6abc	A	10,7 ± 1,2 cd	A	24,7 ± 5,0e	S
*E. punctata* + *E. accedens*	pa	11.0 ± 1.0b	A	18,0 ± 1,7bc	Ad	12,3 ± 2,5ab	A	7,3 ± 1,2ab	A	8,7 ± 2,3abc	A	10,0 ± 0,0ab	A
*E. punctata* + *E. wando*	pw	14.7 ± 1.2c	A	12,7 ± 2,1ab	A	13,0 ± 2,6ab	A	10,0 ± 2,0abc	A	7,3 ± 1,2ab	A	23,30 ± 5,0e	S
*E. accedens* + *E. Robusta*	ar	8.3 ± 0.6ab	A	12,7 ± 0,6ab	A	14,0 ± 2,6ab	A	8,0 ± 0,0ab	A	12,0 ± 0,0de	A	13,3 ± 2,9bcd	A
**Combination of combinations**													
*E. bosistoana* + *E. punctata* + *E accedens* + *E. cladocalyx*	bpca	6.0 ± 0.0a	A	28,7 ± 5,5d	A	23,0 ± 1,7d	S	12,0 ± 0,0	A	7,0 ± 1,7a	A	9,0 ± 0,0ab	A
*E. bosistoana* + *E. lesouefii* + *E. bosistoana* + *E. melliodora*	blbm	7.7 ± 2.1ab	A	20,7 ± 1,2c	A	20,0 ± 0,0 cd	A	12,7 ± 1,2bc	A	8,3 ± 1,5abc	A	7,0 ± 1,0a	A
E. bosistoana + E. melliodora + E. punctata + E. melliodora	bmpm	11.0 ± 1.7b	A	27,7 ± 2,5d	S	10,3 ± 0,6ab	A	11,7 ± 0,6bc	A	6,3 ± 6,3a	A	12,3 ± 1,5bcd	A
E. punctata + E. accedens + E accedens + E. cladocalyx	paac	6.0 ± 0.0a	A	33,3 ± 2,9d	A	10,3 ± 0,6ab	A	10,0 ± 0,0abc	A	6,0 ± 0,0a	A	6,0 ± 0,0a	A
E. punctata + E. wando + E. wando + E. cladocalyx	pwac	6.3 ± 0.6a	A	30,7 ± 1,2d	S	24,0 ± 5,3d	S	15,0 ± 4,0 cd	A	6,0 ± 0,0a	A	9,0 ± 0,0ab	A
E. punctata + E. wando + E. punctata + E. melliodora	pwpm	6.0 ± 0.0a	A	29,0 ± 3,6d	S	15,3 ± 4,2bc	A	11,3 ± 1,2abc	A	6,7 ± 0,6a	A	6,3 ± 0,6a	A
Gentamicine	Genta	24.2 ± 2.3d		31,4 ± 2,1d		38,6 ± 2,8e		21,5 ± 2,4e		26,4 ± 1,6 g		29,9 ± 1,0f	

^*^Ab Abbreviation of Eucalyptus species

^**^Values are means (mm ± MSD) of triplicate determination

^***^Values with different letters differ significantly by Duncan’s multiple range test (*P* < 0.05)

^****^A Antagonism, *Ad* Addition, *S* Synergy

#### Binary combinations

The results indicated that six out of nine binary combinations (bp, ac, bm, pm, pa, and pw) exhibited an increase in activity (Additive and Synergistic) against four bacterial strains (*Haemophilus influenzae*, *Haemophilus parainfluenzae*, *Pseudomonas aeruginosa* ATTC 9027, and *Staphylococcus aureus* ATCC 6538) (Table 1).

Specifically, the binary combination ac showed a synergistic effect against *Pseudomonas aeruginosa* ATCC 9027 and *Staphylococcus aureus* ATCC 6538, while the binary combination bm showed a synergistic effect against *Haemophilus parainfluenzae* and *Pseudomonas aeruginosa* ATCC 9027. The remaining binary combinations displayed an increase in the antibacterial activity against only one bacterial strain each. The bp combination showed a synergistic effect against *Pseudomonas aeruginosa* ATTC 9027; pw—synergistic effect against *Staphylococcus aureus* ATCC 6538. The sole additive effect was observed with the pa combination against *Haemophilus influenzae* (Table 1).

#### Combination of binary combinations

The results of the antibacterial activities of EO mixtures (Table 1) highlighted that four out of six combinations (bpac, bmpm, pwac, and pwpm) demonstrated a synergistic effect against two bacterial strains (*Haemophilus influenzae* and *Haemophilus parainfluenzae*) (Table 1). Notably, the pwac combination displayed a synergistic effect against both bacterial strains, while the combinations bmpm and pwpm exhibited a synergistic effect exclusively against *Haemophilus influenzae*. The remaining bpac combination demonstrated a synergistic effect solely against *Haemophilus parainfluenzae*.

While the majority of EO combinations displayed antagonistic effects against all or several bacterial strains, some exhibited notably higher antibacterial activity compared to individual or binary combined oils. For instance, the observed activity of binary combinations ac and bp against *H. influenzae*, pm, bl against *H. parainfluenzae*, bl against *K. pneumoniae*, and bp against *S. aureus* surpassed the activity of the individual EOs used. Interestingly, certain combinations of binary blends demonstrated even higher activities than those observed with binary EO blends. Notable examples include bpac against *H. influenzae* and blbm against *H. influenzae* and *H. parainfluenzae*.

### Principal Component's analysis (PCA) and Hierarchical Clusters Analysis (HCA) of the antibacterial activity

**Fig. 1:**
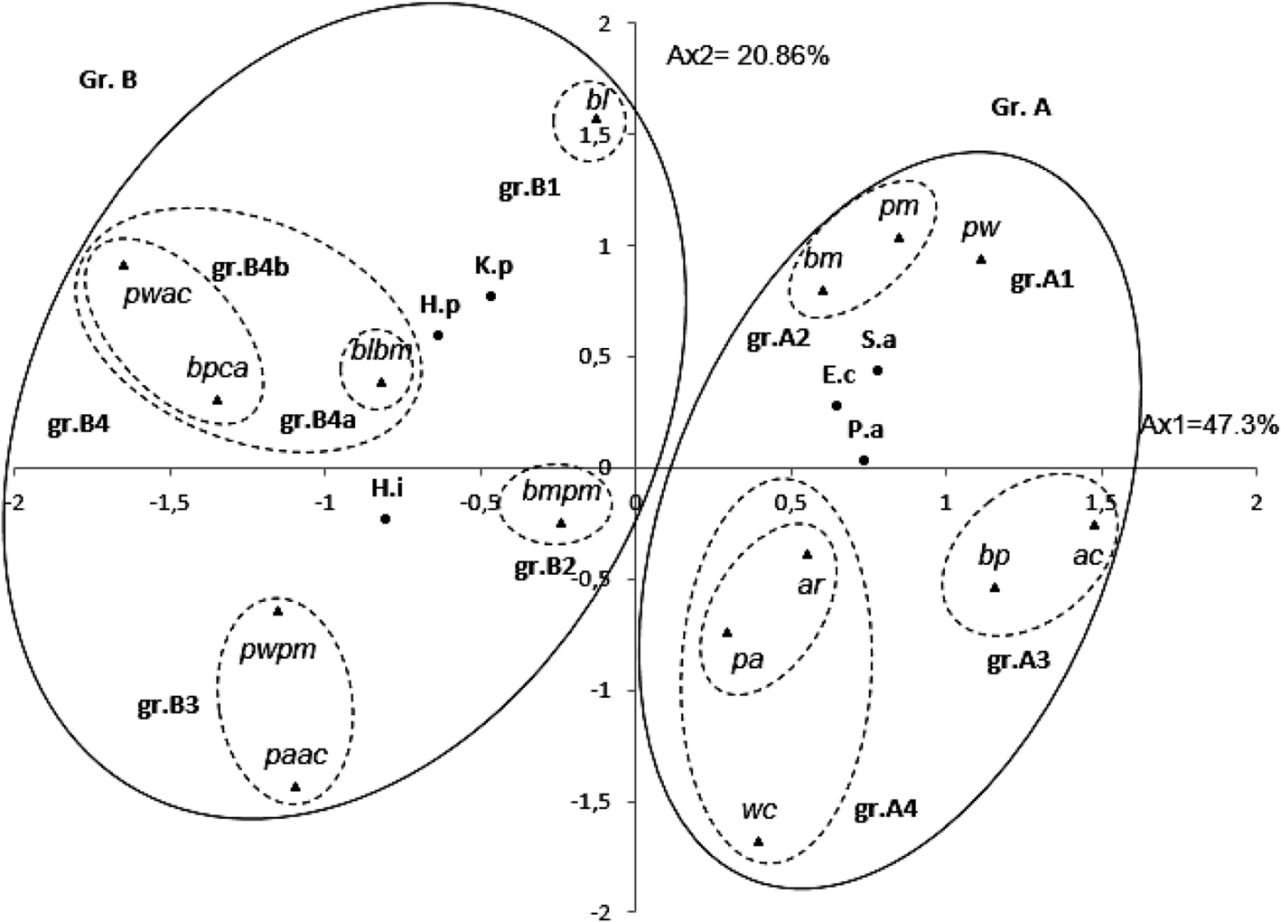
PCA of the antibacterial activities of leaf essential oils of 15 *Eucalyptus* EOs blends obtained from eight Tunisian *Eucalyptus* species. For the abbreviation of the *Eucalyptus* species (▲), see Table 1.*) *E.c: Echerichia coli, k.p: Klebsiella pneumoniae; P.a: Pseudomonas aeruginosa; S.a: Staphylococcus aureus; H.i: Haemophilus influenzae; H.p: Haemophulis parainfluenzae*

**Fig. 2:**
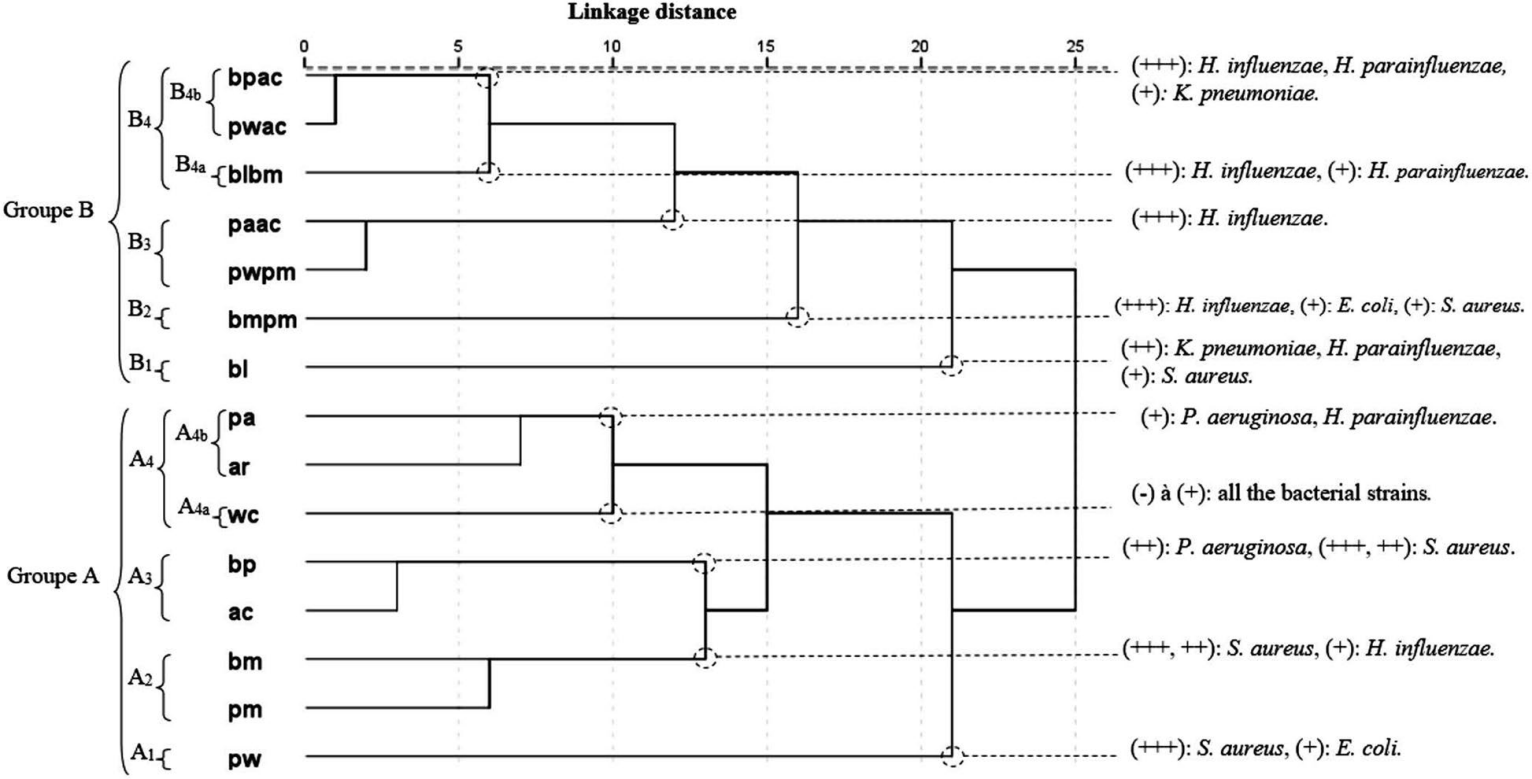
Dendrogram obtained by hierarchical cluster analysis based on the Euclidean distance between groups of the antibacterial activities of 15 EOs blends obtained from leaf essential oils of eight Tunisian *Eucalyptus* species. *) For the abbreviation of the *Eucalyptus* species, see Table 1.*) w: *E. wandoo*; p: *E. punctata*; c: *E. cladocalyx*; a: *E. accedens*; m: *E. melliodora*; b: *E. bosistoana*; l: *E. lesouefii*; r: *E. robusta*

The PCA horizontal axis accounted for 47.30% of the total variance, while the vertical axis contributed an additional 20.96% (Fig. 1). Utilizing HCA based on the Euclidean distances, two distinct groups (A and B) of EO blends were identified by their antibacterial inhibition of growth with a dissimilarity ≥ 22.0% (Fig. 2). Subsequently, when the dissimilarity dropped to ≤ 10%, these groups were further subdivided into four subgroups (A1, A2, A3, A4, B1, B2, B3, and B4).

Within the A4 subgroup, a subsequent division resulted in two subgroups (A4a and A4b) with a dissimilarity ≥ 7.0%. Conversely, the B4 subgroup underwent a division into two subgroups (B4a and B4b) with a dissimilarity < 6.0%. The horizontal axis effectively separated the EO combinations constituting the two main groups (A and B), while the vertical axis facilitated the separation of combined oils within the same groups.

- *Group A*—It consisted of eight binary mixtures: pm, bm, pw, ar, pa, bp, ac, and wc. This group showed a positive correlation with axis 1 and a negative correlation with axis 2. These blends exhibited the strongest antibacterial effect against *E. coli*, *S. aureus*, and *P. aeruginosa*.
  - *Subgroup A1***—**This subgroup comprises a blend of *E. punctata* and *E. wandoo* EOs (pw), displaying a potent inhibitory effect against *S. aureus* (23.30 ± 5.0 mm; izd). Despite exhibiting the highest activity against *E. coli* (14.7 ± 1.2 mm; izd) (Table 1) among all tested combinations, it was interpreted as antagonistic. Notably, this blend showed no activity against *P. aeruginosa*.
  - *Subgroup A2*—This subgroup comprises a blend of *E. punctata* and *E. melliodora* (pm) as well as a blend of *E. bosistoana* and *E. melliodora* (bm). Both combinations share almost the same high activity against *S. aureus* and *H. parainfluenzae*, a mild effect against *K. pneumoniae*, and a low activity against *E. coli*. The separation between the two blends was essentially due to the difference in their activities against *P. aeruginosa*, which was interpreted as an almost moderate activity for bm blend and mild activity for pm blend.
  - *Subgroup A3—*This subgroup contains two binary types, ac and bp. It was distinguished by its moderate activity against *P. aeruginosa*, *S. aureus*, and *H. influenzae* and its mild activity against *E. coli*, *H. parainfluenzae*, and *K. pneumoniae*. The separation between the two binary EO blends was mainly due to the difference in their activities against *S. aureus*, which was interpreted as a strong inhibitory effect of the ac and mild activity for the bp blend oils.
  - *Subgroup A4a—*This subgroup is constituted by the binary combinations ra and pa. These blends of oils showed low activity against *K. pneumoniae* and mild to moderate inhibitory effects against *E. coli*, *P. aeruginosa*, and *H. parainfluenzae* (8.3 ± 0.6 mm ≤ izd ≤ 14.0 ± 2.6 mm). However, they were differentiated by the values of their izd, which were significantly higher with ar blend oil than with pa blend oil, particularly against *P. aeruginosa*, *H. parainfluenzae*, and *S. aureus*; whereas a higher activity was shown for pa against *H. influenzae*.
  - *Subgroup A4b—*This subgroup contains the binary combination wc. It formed a distinct dichotomy with subgroup A4a in the HCA and a separate subgroup in the PCA analyses, with a high negative correlation with axis 2. The wc combination showed the lowest activity against the majority of strains among all combinations.

This subgroup shared with subgroup A4a mild activity against *H. influenzae* and *P. aeruginosa*, *H. influenzae*, and *S. aureus* and the lowest activity against *K. pneumoniae* and *E. coli*.

- *Group B*—This group is formed by the binary mixture of *E. bosistana* and *E. lesouefii* oils (bl) and also by the mixtures of six binary EOs such as bmpm, pwpm, paac, blbm, pwac, and bpac. They were characterized by the strongest activity against *H. influenzae*, *H. parainfluenzae*, and *K. pneumoniae*.
  - *Subgroup B1,* reduced to a binary combination of *E. bosistana* and *E. lesouefii* essential oils (bl), which was clearly separated from the other subgroups of group B essential oil blends in both PCA and HCA analyses. It exhibited the highest activity against *K. pneumoniae* among the tested blends, and a moderate level of activity against *H. parainfluenzae*.
  - *Subgroup B2,* limited to the bmpm EO blend. It exerted a high level of antibacterial activity against *H. influenzae*, almost comparable to that produced by Gentamicine®. However, it was not active against *P. aereuginosa*.
  - *Subgroup B3,* represented by EO blends of pwpm and acpa, were highlighted for their comparable activity to the antibiotic gentamicine against *H. influenzae* (29.0 ± 3.6 and 33.3 ± 2.9 mm; izd; respectively). However, these oils were found to be inactive against *E. coli*, *P. aeruginosa*, and *S. aureus*. They were also grouped together based on their mild antibacterial effect against *K. pneumoniae*. The main difference between these combinations is their activity against *H. parainfluenzae* (10.3 ± 0.6 and 15.3 ± 4.2 mm; izd; respectively).
  - *Subgroup B4* is formed by three EO blends: bpac, pwac, and bmbl. These blends are characterized by strong activity against *H. influenzae* and *H. parainfluenzae*, with izd varying from 20.0 ± 0.0 to 30.7 ± 1.2 mm. However, their activity is still lower than that produced by the antibiotic gentamicine. A synergistic effect is observed with the pwac blend against both strains, and with the bpac against *H. parainfluenzae* only. Despite the antagonistic effect produced by the association of bpac essential oils with *H. influenzae*, the activity is still classified as strong (28.7 ± 5.5 mm, izd), comparable to that produced by gentamicin (31.4 ± 2.1 mm, izd). A moderate level of activity was shown for pwac, bpac, and bmbl combinations (15.0 ± 4.0, 12.0 ± 0.0 and 12.7 ± 1.2 mm; izd; respectively) against *K. pneumoniae*.

### Broth microdilution method

The results of the MIC and MBC of the tested EO blends are presented in Table 2. They demonstrated bactericidal effects against the bacterial strains tested, with MBC/MIC values less than 4, except for the pm blend against *K. pneumoniae*, the mbpm blend against *S. aureus*, and the paac blend against *P. aeruginosa*, *E. coli*, and *H. influenzae*. The binary EO combinations pa and pw exhibited the strongest antibacterial activity against all tested bacterial strains.

**Table 2:** Minimal inhibitory concentration (MICs), minimal bactericidal concentrations (MBC s), and MBC/MIC ratio for 15 blends Eos extracted from leaves of eight Eucalyptus *Species*

Com*)	Souches bactériennes																	
	Gram-negatif															Gram-positif		
	*Echerichia coli*			*Haemophilus influenzae*			*Haemophilus parainfluenzae*			*Klebsiella pneumoniae*			*Pseudomonas aeruginosa*			*Staphylococcus aureus*		
	CMI	CMB	CMB/CMI	CMI	CMB	CMB/CMI	CMI	CMB	CMB/CMI	CMI	CMB	CMB/CMI	CMI	CMB	CMB/CMI	CMI	CMB	CMB/CMI
lb**)	103,9	103,9	1,0	207,7	207,7	1,0	103,9	103,9	1,0	51,9	51,9	1,0	207,7	207,7	1,0	103,9	103,9	1,0
bp	103,9	103,9	1,0	415,5	-	0,0	103,9	103,9	1,0	103,9	103,9	1,0	415,5	-	NT	207,7	207,7	1,0
pa	14,1	14,1	1,0	103,9	103,9	1,0	103,9	103,9	1,0	6,5	6,5	1,0	207,7	207,7	1,0	103,9	103,9	1,0
wc	51,9	51,9	1,0	51,9	103,9	2,0	103,9	103,9	1,0	51,9	51,9	1,0	103,9	103,9	1,0	103,9	103,9	1,0
pw	14,1	14,1	1,0	51,9	103,9	2,0	0,8	1,6	2,0	14,1	26,0	2,0	103,9	103,9	1,0	51,9	51,9	1,0
ar	51,9	51,9	1,0	415,5	-	0,0	415,5	-		207,7	207,7	1,0	103,9	103,9	1,0	207,7	207,7	1,0
bm	103,9	103,9	1,0	103,9	103,9	1,0	103,9	207,7	2,0	51,9	51,9	1,0	207,7	207,7	1,0	207,7	207,7	1,0
pm	103,9	103,9	1,0	103,9	207,7	2,0	207,7	207,7	1,0	0,8	3,3	4,0	103,9	103,9	1,0	103,9	103,9	1,0
ac	415,5	-	-	103,9	103,9	1,0	103,9	103,9	1,0	103,9	103,9	1,0	415,5	-	0,0	207,7	207,7	1,0
bpac	103,9	207,7	2,0	103,9	207,7	2,0	103,9	207,7	2,0	103,9	207,7	2,0	207,7	415,5	2,0	207,7	207,7	1,0
pwac	207,7	207,7	1,0	207,7	415,5	2,0	207,7	415,5	2,0	103,9	207,7	2,0	207,7	207,7	1,0	207,7	415,5	2,0
pwpm	103,9	207,7	2,0	207,7	207,7	1,0	207,7	415,5	2,0	103,9	207,7	2,0	207,7	415,5	2,0	103,9	207,7	2,0
bmpm	103,9	207,7	2,0	207,7	415,5	2,0	207,7	415,5	2,0	207,7	207,7	1,0	103,9	207,7	2,0	103,9	415,5	4,0
paac	103,9	415,5	4,0	103,9	415,5	4,0	51,9	103,9	2,0	103,9	207,7	2,0	103,9	415,5	4,0	415,5	415,5	1,0
blbm	207,7	415,5	2,0	415,5	415,5	1,0	207,7	415,5	2,0	103,9	207,7	2,0	415,5	415,5	1,0	207,7	415,5	2,0
Gent*)	0,6	2,5	4,2	6,0	24,0	4,0	6,0	24,0	4,0	4,0	16,0	4,0	10,0	10,0	1,0	5,0	20,0	4,0

The pm and pw combinations showed the lowest MIC and MBC against *K. pneumoniae* and *H. parainfluenzae* (0.8 mg/ml, 3.3 mg/ml and 0.8 mg/ml, 1.6 mg/ml, respectively). The pa blend also showed low MIC and MBC against *K. pneumoniae* (6.5 mg/ml) and *E. coli* (14.1 mg/ml), whereas the pm blend exhibited similar results as the pa blend.

The highest MIC value (415.5 mg/ml) was observed for the binary mixture of bp, ac, and blbm against *P. aeruginosa*. However, the highest MIC results were observed with the blend of ar against *H. influenzae* and *H. parainfluenzae*, the paac blend against *S. aureus*, and the ac blend against *E. coli*.

### Fraction inhibitory concentration index

Based on the FIC index (Table 3), the results showed that among the 90 antibacterial effects observed with 15 *Eucalyptus* EO blends, 6 (6.66%) showed total synergism, 1 (1.11%) showed partial synergism, 11 (12.22%) had additive effects, 50 (55.5%) had indifferent effects, and 22 (24.44%) had antagonistic effects.

**Table 3:** FIC_I_ values of 15 Blends EOs of eight Eucalyptus species against six bacterial strains responsible for ear infection

EOS blends	Bacterial strains					
	*E. coli*	*H. influenzae*	*H. parainfluenzae*	*K. pneumoniae*	*P. aeruginosa*	*S. aureus*
bl*)	2,3^d^	2,5^d^	0,8^c^	0,7^b^	1,0^c^	1,3^d^
bp	4,0^d^	8,0^e^	1,0^c^	2,0^d^	2,0^d^	4,0^d^
pa	0,2^a^	1,5^d^	1,5^d^	0,2^a^	3,0^d^	1,3^d^
wc	1,0^c^	17,2^e^	2,5^d^	2,0^d^	2,5^d^	3,0^d^
pw	0,3^a**)^	1,5^d^	tr^a^	0,4^a^	3,0^d^	1,5^d^
ar	3,8^d^	6,0^e^	8,0^e^	8,0^e^	1,0^c^	1,5^d^
bm	6,0^e^	2,0^d^	1,0^c^	2,5^d^	1,5^d^	4,0^d^
pm	5,0^e^	2,0^d^	2,0^d^	0,2^a^	1,5^d^	2,0^d^
ac	5,0^e^	33,0^e^	1,5^d^	4,0^d^	4,0^d^	2,5^d^
bpac	1,3^d^	1,3^d^	2,0^d^	2,0^d^	1,0^c^	2,0^d^
pwac	14,8^e^	4,0^d^	262,1^e^	7,4^e^	2,0^d^	4,0^d^
pwpm	8,4^e^	6,0^e^	260,6^e^	137,2^e^	4,0^d^	3,0^d^
bmpm	2,0^d^	4,0^d^	4,0^d^	8,0^e^	1,0^c^	1,0^c^
paac	7,6^e^	2,0^d^	1,0^c^	17,0^e^	0,8^c^	6,0^e^
blbm	4,0^d^	6,0^e^	4,0^d^	4,0^d^	4,0^d^	3,0^e^

^*^l: *E. lesouefii*; b: *E. bosistoana*; p: *E. punctata*; a: *E. accedens*; c: *E. cladocalyx*; W: *E. wandoo*; r: *E. robusta*; m: *E. melliodora*

^**^a Total synergistic effect (ICFI ≤ 0,5); b Partiel synergistic effect (0,5 < ICFI ≤ 0,75); c Additive effect (0,75 ≤ ICFI ≤ 1);d Indifferent effect (1 ≤ ICFI ≤ 4); e Antagonistic effect(ICFI > 4)

#### Binary combinations

The results of the antibacterial activities of EO mixtures indicated that seven (pa, pw, pm, bl, bp, ar, and wc) out of nine binary combinations showed an increase in activity (total synergism, partial synergism, additive effect) against three bacterial strains (*E. coli*, *H. parainfluenzae*, and *K. pneumoniae*) (Table 1).

The pw blend oils demonstrated the best antibacterial activity, displaying total synergism against *H. parainfluenzae*, *E. coli*, and *K. pneumoniae* (FIC_I_ of traces, 0.3, and 0.4, respectively). Additionally, the pa blend showed the highest activity against *E. coli* and *K. pneumoniae* (FIC_I_ = 0.2 for each). The later bacterial strain was sensitive to pm (FIC_I_ = 0.2).

The lb blend showed a partial synergistic effect against *K. pneumoniae* (FIC_I_ = 0.7), while the bp, ar, and wc blends showed an additive effect against *H. parainfluenzae*, *P. aeruginosa*, and *E. coli*, respectively (Table 3).

#### Combination of binary combinations

The results of the study showed that none of the binary combinations had a synergistic effect. However, a significant additive effect was observed for bpac and bmpm against Gram negative bacteria such as *P. aeruginosa*; for paac against *H. parainfluenzae*; and for bmpm against Gram-positive bacteria such as *S. aureus* (Table 3).

### Chemical composition of tested EOs

Chromatographic analysis (GC-RI and GC/MS) of the individual essential oils allowed the identification of 138 components, representing 84.6% to 98.7% of the total oil content [11]. Twenty-one major components were selected based on their qualitative and quantitative addition to the essential oil blends, with an average greater than 0.8% in at least one blend (Table 4). The chemical composition of the oil blends was significantly different between blends (*p* ≤ 0.001), except for *α*-phellandrene, epiglobulol, endo-borenol, spathulenol, and globulol. The identified components were divided into fifteen classes (Table 3), with the main component being the monoterpenic oxide *1,8*-cineole (**1**; 23.7 ± 5.9–59.5 ± 3.8%). The second major class was the monoterpene hydrocarbons (13.3 ± 2.6–46.6 ± 8.2%), with *p*-cymene (**2**; 2.2 ± 0.1–32.3 ± 2.5%), *α*-pinene (**3**; 5.2 ± 0.6–26.7 ± 7.8%), and *β*-pinene (tr-5.6 ± 0.1%) having the highest content. The third major class was the sesquiterpenic alcohols (2.6 ± 0.6–15.2 ± 1.0%), represented essentially by globulol (**4**; 0.1 ± 0.1–7.6 ± 1.6%), spathulenol (0.5 ± 0.5–4.4 ± 2.6%) and viridiflorol (**5**; 0.2 ± 0.0–1.8 ± 0.4%). The monoterpenic alcohols (3.7 ± 0.5–12.2 ± 0.9%) were the fourth major class, represented by *trans*-pinocarveol (2.1 ± 0.2–4.3 ± 0.9%) and *α*-terpineol (0.3 ± 0.1–3.6 ± 0.1%). The fifth class was composed of sequiterpene hydrocarbons (1.1 ± 0.2–8.2 ± 0.8%), essentially by aromadendrene (**6**; 0.1 ± 0.0–4.4 ± 0.3%). Cryptone (0.1 ± 0.0–4.3 ± 1.0%) was the principal compound of the monoterpenic ketones (0.7 ± 0.2–6.6 ± 1.4%), while methyl amyl acetate (tr-4.5 ± 0.8%) was the main aliphatic ester (traces-4.5 ± 0.8%). The aldehydes, phenols, and other minor compound classes were not discussed.

**Table 4:** Content [%] of the 21 Compounds selected for the Principal Component and the Hierarchical Cluster Analyses in 15 blends of EOs Extracted from the Leafs of eight Eucalyptus species and their chemical classes

Compounds	Abreviation	Content [%]														
		ac*)	ar	bl	blmb	bm	bmpm	Bp	bpac	pa	paac	pm	pw	pwac	pwpm	wc
*α*-Pinene	α-pin	21,0 ± 6.5	26,7 ± 7.8	11,8 ± 2.2	10,9 ± 2.0	10.0 ± 1.9	8,5 ± 1.4	7.8 ± 3.6	14.4 ± 5.1	21,5 ± 8.3	21,3 ± 0.9	7,0 ± 1.0	5,7 ± 1.2	13,4 ± 3.8	6,4 ± 1.1	5,2 ± 0.6
*β*-Pinene	Β-pin	Tr^b)^	0,1 ± 0.1	5,6 ± 0.1	2,9 ± 0.1	0,2 ± 0.1	1,6 ± 0.7	3.2 ± 1.5	1.6 ± 0.7	3,0 ± 1.4	1,5 ± 0.7	3,1 ± 1.4	3,1 ± 1.4	1,5 ± 0.7	3,1 ± 1.4	0,1 ± 0.0
*α*-Phellandrene	α-phe	1,1 ± 0.9	1,2 ± 0.9	0,2 ± 0.1	0,2 ± 0.0	0,2 ± 0.0	0,2 ± 0.0	0.2 ± 0.1	0.7 ± 0.4	1,2 ± 0.9	1,2 ± 0.0	0,2 ± 0.2	0,1 ± 0.0	0,6 ± 0.4	0,2 ± 0.2	tr
*p*-Cymene	p-cym	4,5 ± 1.5	10,2 ± 1.5	5,9 ± 0.3	4,0 ± 0.2	2,2 ± 0.1	8,4 ± 0.1	16.4 ± 0.6	10.5 ± 0.4	18,7 ± 1.1	11,6 ± 0.2	14,6 ± 0.1	32,3 ± 2.5	18,4 ± 0.5	23,5 ± 1.3	18,1 ± 2.1
*1,8*-Cineole	1,8-cin	33,6 ± 5.5	27,3 ± 2.9	45,3 ± 4.0	52,4 ± 3.9	59,5 ± 3.8	51,2 ± 0.2	36.0 ± 1.5	34.8 ± 2.0	23,7 ± 5.9	28,7 ± 0.2	42,8 ± 3.4	28,6 ± 5.3	31,1 ± 5.4	35,7 ± 4.3	38,5 ± 4.9
*γ*-Terpinene	γ-ter	0,1 ± 0.0	0,2 ± 0.0	0,2 ± 0.0	0,1 ± 0.0	0,1 ± 0.0	0,1 ± 0.0	0.1 ± 0.0	0.1 ± 0.0	0,1 ± 0.0	0,1 ± 0.0	0,1 ± 0.0	2,0 ± 0.9	1,0 ± 0.4	1,0 ± 0.4	2,0 ± 0.9
*trans*-Pinocarveol	tr-pin	2,2 ± 0.4	3,8 ± 0.5	3,1 ± 0.2	3,3 ± 0.2	3,5 ± 0.1	3,9 ± 0.4	3.8 ± 0.7	3.0 ± 0.2	3,4 ± 0.5	2,8 ± 0.5	4,3 ± 0.9	3,4 ± 0.7	2,8 ± 0.1	3,9 ± 3.8	2,1 ± 0.2
citronellal	cit	0,2 ± 0.0	1,8 ± 0.0	0,5 ± 0.0	0,3 ± 0.0	0,1 ± 0.0	0,1 ± 0.0	0.1 ± 0.0	0.2 ± 0.0	0,1 ± 0.0	0,2 ± 0.0	0,1 ± 0.0	0,1 ± 0.0	0,2 ± 0.0	0,1 ± 0.0	0,2 ± 0.0
*endo*-Borneol	enb	0,4 ± 0.1	3,2 ± 0.2	0,7 ± 0.0	0,7 ± 0.0	0,8 ± 0.0	0,9 ± 0.1	1.1 ± 0.2	0.7 ± 0.1	0,9 ± 0.2	0,6 ± 0.1	1,0 ± 0.3	0,8 ± 0.2	0,6 ± 0.1	0,9 ± 0.3	0,3 ± 0.0
Terpinen-4-ol	Ter-4-ol	0,3 ± 0.1	0,3 ± 0.1	1,8 ± 0.0	1,0 ± 0.0	0,3 ± 0.0	0,3 ± 0.0	0.3 ± 0.1	03 ± 0.1	0,4 ± 0.1	0,3 ± 0.0	0,4 ± 0.1	0,8 ± 0.1	0,5 ± 0.0	0,6 ± 0.0	0,7 ± 0.2
Cryptone	cry	0,2 ± 0.1	0,1 ± 0.0	0,7 ± 0.1	0,5 ± 0.1	0,3 ± 0.1	2,1 ± 0.5	4.3 ± 1.0	2.3 ± 0.5	4,0 ± 1.0	2,1 ± 0.5	4,0 ± 1.0	4,1 ± 1.0	2,2 ± 0.5	4,0 ± 1.0	0,3 ± 0.0
*α* -Terpineol	α–ter	0,3 ± 0.1	3,6 ± 0.1	1,1 ± 0.1	1,2 ± 0.0	1,3 ± 0.0	1,2 ± 0.1	0.8 ± 0.1	0.5 ± 0.1	0,5 ± 0.21	0,4 ± 0.0	1,1 ± 0.1	0,9 ± 0.3	0,6 ± 0.2	1,0 ± 0.2	0,7 ± 0.2
*p*-Cymen-8-ol	p-cy-8-ol	0,1 ± 0.0	0,3 ± 0.0	0,1 ± 0.0	0,1 ± 0.0	0,1 ± 0.0	0,9 ± 0.4	1.7 ± 0.7	0.9 ± 0.4	1,8 ± 0.7	0,9 ± 0.3	1,7 ± 0.7	1,7 ± 0.7	0,9 ± 0.4	1,7 ± 0.7	0,1 ± 0.0
Cuminaldehyde	cum	tr	0,1 ± 0.0	0,1 ± 0.0	tr	tr	0,5 ± 0.2	1.0 ± 0.4	0.5 ± 0.2	1,0 ± 0.4	0,5 ± 0.2	1,0 ± 0.4	1,0 ± 0.4	0,5 ± 0.2	1,0 ± 0.4	tr
Aromadendrene	aro	2,6 ± 2.8	0,2 ± 0.1	3,8 ± 1.8	3,9 ± 1.8	3,9 ± 1.7	2,1 ± 0.8	3.7 ± 1.9	4.1 ± 0.8	0,1 ± 0.1	2,3 ± 0.2	0,3 ± 0.1	0,1 ± 0.0	2,3 ± 0.1	0,2 ± 0.0	4,4 ± 0.3
epiglobulol	epi	0,8 ± 0.8	0,2 ± 0.0	0,5 ± 0.6	0,5 ± 0.6	0,5 ± 0.6	0,3 ± 0.3	0.5 ± 0.6	0.8 ± 0.2	0,1 ± 0.0	0,6 ± 0.1	0,1 ± 0.0	0,1 ± 0.0	0,6 ± 0.1	0,1 ± 0.0	1,2 ± 0.2
Spathulenol	spa	0,5 ± 0.5	1,3 ± 1.4	4,4 ± 2.6	3,4 ± 2.7	2,3 ± 2.7	1,7 ± 1.4	2.8 ± 2.8	2.1 ± 2.2	2,0 ± 1.7	1,7 ± 0.1	1,0 ± 0.0	0,8 ± 0.1	1,0 ± 0.8	0,9 ± 0.1	0,1 ± 0.0
Globulol	glo	7,6 ± 1.6	1,9 ± 0.1	1,6 ± 0.4	1,7 ± 0.5	1,8 ± 0.6	1,2 ± 0.4	1.2 ± 0.4	4.4 ± 0.1	1,2 ± 0.2	4,4 ± 0.7	0,6 ± 0.2	0,1 ± 0.1	3,8 ± 0.8	0,3 ± 0.1	6,4 ± 1.4
Viridiflorol	vir	1,8 ± 0.4	0,6 ± 0.1	0,4 ± 0.1	0,4 ± 0.1	0,5 ± 0.1	0,3 ± 0.0	0.4 ± 0.1	1.1 ± 0.2	0,5 ± 0.1	1,2 ± 0.1	0,2 ± 0.0	0,1 ± 0.0	1,0 ± 0.2	0,2 ± 0.0	1,4 ± 0.3
Rosifoliol	ros	1,0 ± 0.0	2,7 ± 0.3	0,2 ± 0.2	0,2 ± 0.2	0,2 ± 0.2	0,2 ± 0.1	0.3 ± 0.2	0.6 ± 0.0	0,2 ± 0.1	0,6 ± 0.1	0,1 ± 0.0	0,1 ± 0.0	0,5 ± 0.1	0,1 ± 0.0	0,9 ± 0.2
Methyl amyl acetate	maa	4,5 ± 0.8	tr	tr	tr	tr	tr	tr	2.2 ± 0.4	tr	2,2 ± 0.4	tr	tr	2,2 ± 0.4	tr	4,5 ± 0.8
Chemicals classes																
Monterpens fydrocarbons		27,9 ± 4,6	40,7 ± 5.0	24,8 ± 2.8	19,1 ± 2.7	13,3 ± 2.6	19,8 ± 2.7	29,0 ± 6.1	28,5 ± 5.4	46,6 ± 8.2	37,3 ± 1.8	26,3 ± 2.8	44,6 ± 6.4	36,3 ± 5.5	35,5 ± 4.6	25,9 ± 2.8
Monoterpenic oxydes		33,7 ± 5.5	27,7 ± 2.4	45,4 ± 4.0	52,5 ± 3.9	59,6 ± 3.8	51,4 ± 0.1	36,4 ± 1.3	35,1 ± 2.1	24,1 ± 6.0	28,9 ± 0.2	43,2 ± 3.5	28,9 ± 5.4	31,3 ± ± 5.5	36,1 ± 4.5	38,5 ± 4.9
Monoterpenic ketones		0,7 ± 0.2	0,8 ± 0.1	1,6 ± 0.2	1,6 ± 0.1	1,5 ± 0.1	4,0 ± 0.7	6,6 ± 1.4	3,6 ± 0.8	6,2 ± 1.3	3,4 ± 0.5	6,5 ± 1.2	6,3 ± 1.3	3,5 ± 0.7	6,4 ± 1.2	0,7 ± 0.2
Monoterpenic aldehydes		0,3 ± 0.0	2,1 ± 0.1	0,8 ± 0.0	0,5 ± 0.0	0,2 ± 0.0	1,0 ± 0.3	1,8 ± 0.6	1,1 ± 0.3	1,8 ± 0.6	1,0 ± 0.3	1,8 ± 0.6	1,7 ± 0.6	1,0 ± 3.0	1,8 ± 0.6	0,3 ± 0.0
Monoterpenic alcohols		3,7 ± 0.5	12,2 ± 0.9	8,4 ± 0.3	8,0 ± 0.2	7,5 ± 0.2	8,6 ± 0.7	7,9 ± 1.2	5,8 ± 0.3	7,1 ± 0.8	5,4 ± 0.7	9,6 ± 1.6	7,7 ± 1.5	5,7 ± 0.5	8,7 ± 1.6	4,3 ± 0.2
Phenols		0,2 ± 0.1	0,5 ± 0.1	0,3 ± 0.0	0,2 ± 0.0	0,2 ± 0.0	0,4 ± 0.0	0,7 ± 0.0	0,4 ± 0.0	0,7 ± 0.0	0,5 ± 0.0	0,7 ± 0.1	1,0 ± 0.0	0,6 ± 0.0	0,8 ± 0.0	0,4 ± 0.1
Monetrpenic esters		0,4 ± 0.1	1,8 ± 0.1	0,6 ± 0.0	0,8 ± 0.0	1,0 ± 0.0	1,9 ± 0.4	2,2 ± 0.7	1,3 ± 0.3	2,1 ± 0.7	1,3 ± 0.4	2,8 ± 0.7	2,3 ± 0.7	1,4 ± 0.3	2,6 ± 0.7	0,6 ± 0.1
Methyl ether phenols		-	tr	tr	tr	tr	tr	tr	tr	-	-	tr	tr	tr	tr	tr
Hydrocarboncic sesquiterpens		8,2 ± 0.8	1,6 ± 0.1	5,8 ± 2.4	6,9 ± 2.3	7,9 ± 2.2	5,4 ± 1.0	5,4 ± 2.5	6,8 ± 0.9	1,1 ± 0.2	4,7 ± 0.5	2,8 ± 0.2	0,3 ± 0.1	4,3 ± 0.4	1,6 ± 0.1	7,4 ± 0.9
Sesquiterpenic alcohols		15,2 ± 1.0	8,5 ± 1.5	8,6 ± 3.5	7,4 ± 3.5	6,3 ± 3.4	4,5 ± 1.5	6,0 ± 3.7	10,6 ± 1.4	5,6 ± 1.5	10,4 ± 1.3	2,6 ± 0.3	2,6 ± 0.8	8,9 ± 0.9	2,6 ± 0.6	12,2 ± 3.3
Sesquiterpenic oxydes		-	-	-	-	-	0,4 ± 0.1	0,7 ± 0.2	0,4 ± 0.1	0,7 ± 0.2	0,4 ± 0.1	0,7 ± 0.2	0,7 ± 0.2	0,4 ± 0.1	0,7 ± 0.2	-
Sesquiterpenic aldehydes		tr	-	tr	tr	tr	tr	tr	tr	-	tr	-	-	tr	-	tr
Oxygenated aromatics		tr	tr	tr	tr	tr	tr	tr	tr	tr	tr	tr	tr	tr	tr	tr
Ketones		tr	tr	tr	tr	tr	tr	tr	tr	tr	tr	tr	tr	tr	tr	tr
Aliphatic esters		4,5 ± 08	tr	tr	tr	tr	tr	tr	2,2 ± 0.4	tr	2,2 ± 0.4	tr	tr	2,2 ± 0.4	tr	4,5 ± 0.8

^*^The abbreviation of the *Eucalyptus* species are given in Table 1.^**^tr Trace (< 0.1%).^***^Not detected

### Chemical Principal Component's analysis (PCA) and Hierarchical Clusters Analysis (HCA)

**Fig. 3:**
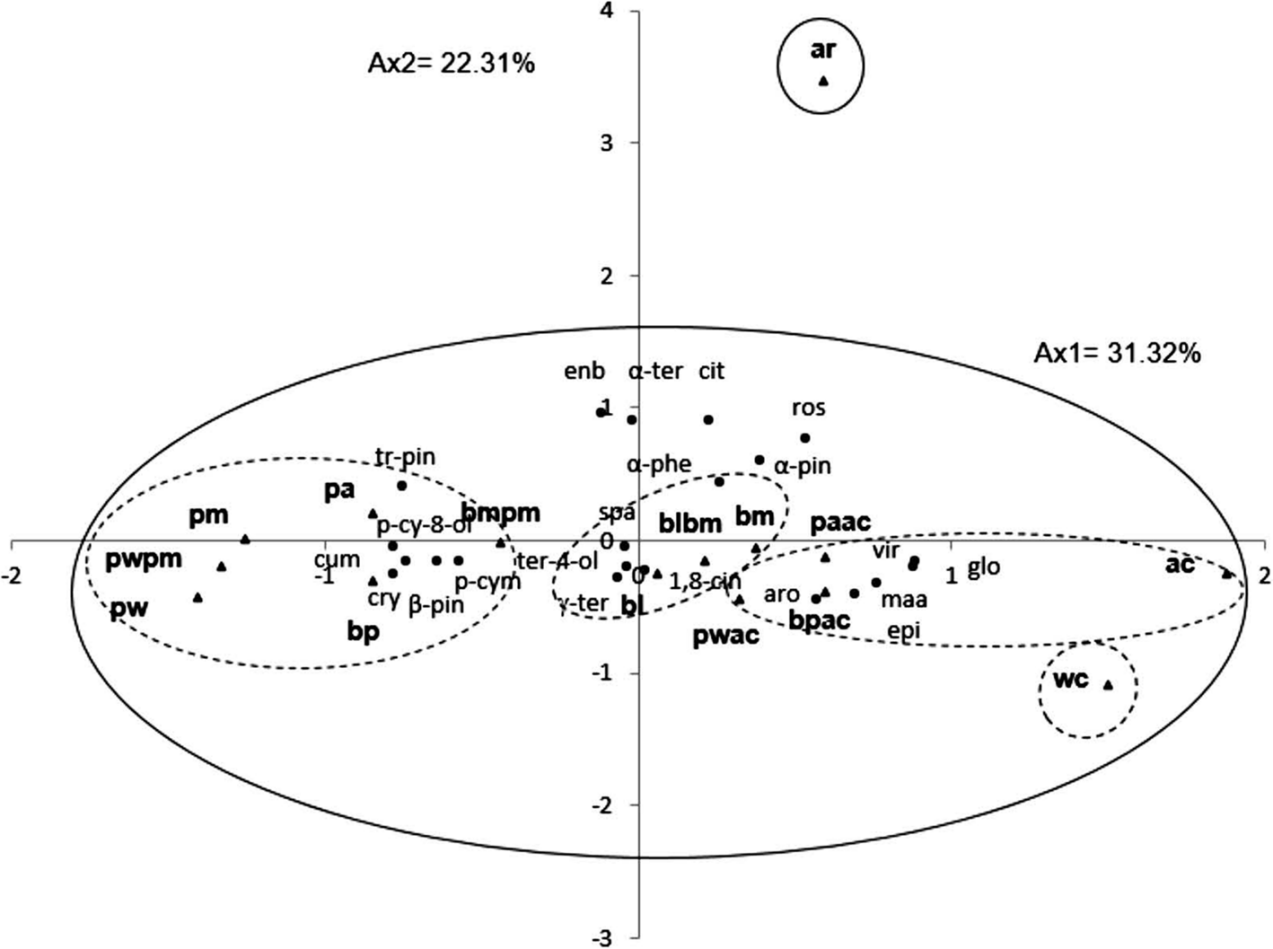
PCA of twenty one components for the 15 *Eucalyptus* EOS blends extracted from leaf of eight Tunisian *Eucalyptus* species. For the abbreviation of the *Eucalyptus* species (▲), see Table 1

**Fig. 4:**
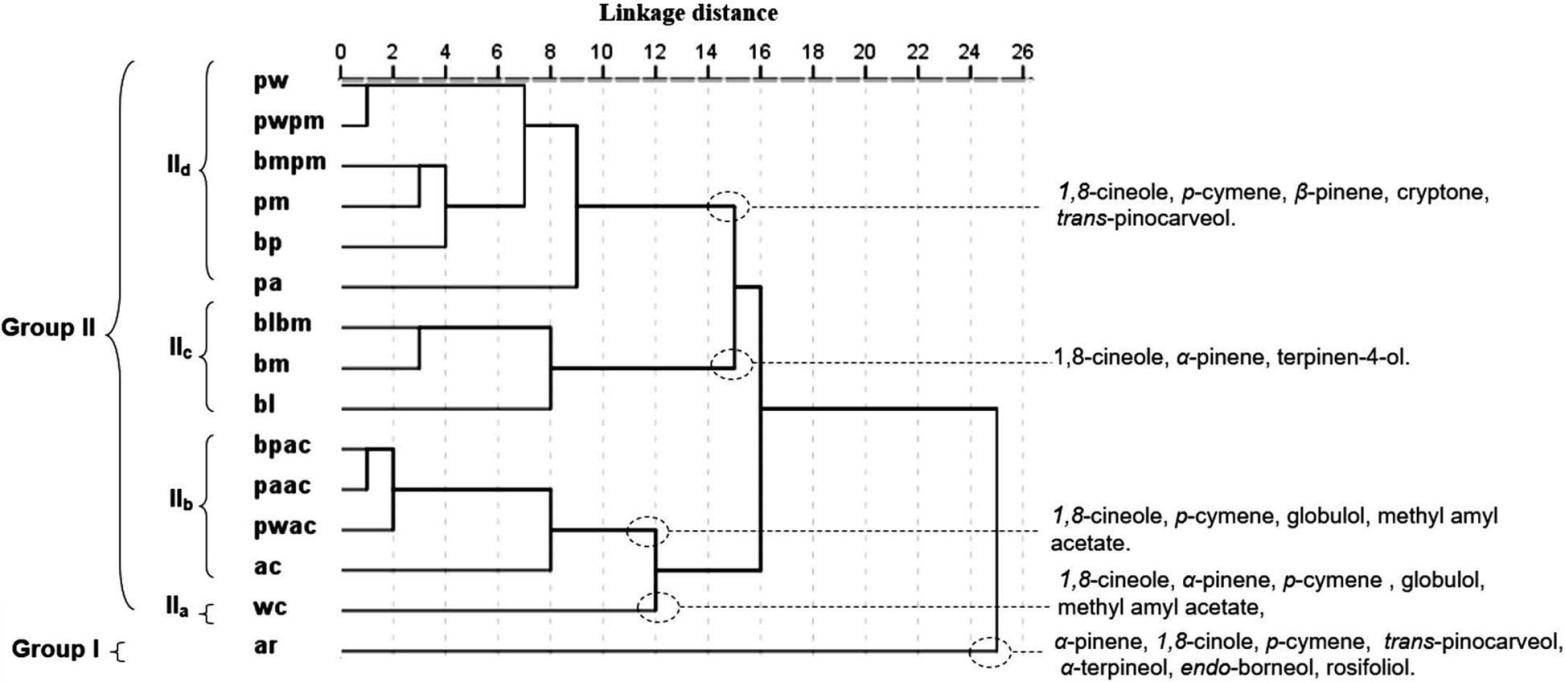
Dendrogram obtained by hierarchical cluster analysis based on the Euclidean distance between groups of 15 *Eucalyptus* blends EOs extracted from leaf of eight Tunisian *Eucalyptus* species. Components that characterize the major subgroups, considered as chemotypes, are indicated. For the abbreviation of the *Eucalyptus* species (▲), see Table 1

In order to investigate the relationship between the chemical composition and antibacterial activity of EO blends, 21 selected oil blend components listed in Table 4 were analyzed using PCA and HCA. The PCA analysis showed that the horizontal axis explained 31.32% of the total variance, while the vertical axis explained 22.31% (Fig. 3). The HCA analysis revealed two distinct groups of essential oil blends (I and II) with a dissimilarity score greater than 17 (Fig. 4). Axis 1 was responsible for separating the essential oil blends of group I from those of group II, while axis 2 was responsible for separating EO blends within group II (Fig. 4).

*Group I* was reduced to the mixture of *E. astringens* and *E. robusta* oils (ar) and showed a positive correlation with both axes, forming a separate group in PCA and a deep dichotomy in HCA. This group was distinguished by having the highest mean percentages of *α*-pinene (26.7 ± 7.8%), *trans*-pinocarveol (3.8 ± 0.5%), α-terpineol (3.6 ± 0.1%), *endo*-borneol (3.2 ± 0.2%), and rosifoliol (2.7 ± 0.3%). However, it was relatively rich in p-cymene but poor in *1,8*-cineole.

*Group II* showed a high correlation with axis 1 and a low correlation with axis 2. With a dissimilarity index greater than 9, it was separated into four subgroups: IIa, IIb, IIc, and IId.

*Subgroup IIa*, limited to the mixture of *E. wandoo* and *E. cladocalyx* oils (Wc). It shared with group IIb (ac, pwac, paca, and bpac) the highest mean percentage in globulol, methyl amyl acetate, aromadedrene, viridiflol [7], and epiglobulol, as well as medium percentages in *1,8*-cineole, *α*-pinene, and *p*-cymene, and the lowest content in *β*-pinene. The separation between the EO blends of the two groups was mainly due to the variation in their chemical component content. In fact, the wc blend oils were very close to the ac blend oils in both the HCA and the PCA, which could be explained by their similar high mean percentages of globulol and methyl amyl acetate. However, they were separated by the increasing level of p-cymene in the wc blend oils and its decreasing level in the ac blend oils, which were significantly richer in *α*-pinene. This compound was identified as characterizing the rest of group IIb blends, with a mean percentage varying between 21.3 ± 0.9% and 13.4 ± 3.8%.

*Subgroup IIc*, formed by the EO blends of bl, bm, and blbm. These oils are distinguished by their highest levels of *1,8*-cineole, spathulenol, terpinene-4-ol, and α-terpineol, as well as their relatively high contents of α-pinene and trans-pinocarveol. However, they are relatively poor in p-cymene, rosifoliol, methyl amyl acetate, and cuminaldehyde. The PCA and HCA analyses showed that bm and blbm blends were similar in their disposition, with the highest content of 1,8-cineole and low content of *β*-pinene, compared to lb blend oil, which is richer in spathulenol.

*Subgroup IId*, constituted by pa, bp, pm, bmpm, pwpm, and pw blends. Their EOs were characterized by the highest mean percentage in *p*-cymene, trans-pinocarveol, cryptone, p-cymen-8-ol, and a relatively high mean percentage in β-pinene. With dissimilarity greater than 7, pa blend oils formed a deep dichotomy within the rest of the subgroup IIb blends oils in the HCA analysis. This separation was essentially explained by its disposition, the lowest mean percentage in 1,8-cineole and relatively the highest content in α-pinene. On the other hand, the differentiation of bp, pm, bmpm, pwpm, and pw blends was primarily affected by their content in *1,8*-cineole and p-cymene. The pw and pwpm blends of oils had the highest content in p-cymene, which distinguished them from the other blends, bp, bmpm, and pm, which had relatively higher content in 1,8-cineole. This separation was evident in both principal component analysis (PCA) and HCA, where pw and pwpm blends oils correlated negatively with axis 1 and were clearly separated from the other blends.

## Discussion

The antibacterial activities of EOs are often attributed to the complexity of their chemical composition as well as to the synergistic, additive, or antagonistic effects of their chemical components [19]. Therefore, we tried to enhance the antibacterial activity by mixing EOs. In general, the antibacterial activity of the combined EOs varied significantly according to the chemical composition, the bacterial strains, and the method used. To find a logical explanation for this variation, we based our analysis on the principle of the quantitative and qualitative addition of the main compounds constituting the EO blends (Table 4) and their correlations with the observed activity.

### Correlation of the antibacterial activity of EO blends tested by the disc diffusion method with their chemical compositions

It was reported that the EO's biological and pharmacological activity is attributed to the effect of the complex interaction between the EO components' functional groups belonging to some chemical classes such as phenols, alcohols, esters, ethers, and to their mean percentages and the bacterial strains [8, 20, 21].

### Antibacterial activity against Gram (-) bacterial strains

#### Haemophilus influezae

The highest effect of the EO combinations was observed, especially with the mixtures of paac and pwac oil blends (33.3 ± 2.9 and 30.7 ± 1.2 mm; izd; respectively) (Table 1). These EO blends were distinguished by the highest mean percentages of globulol (4.4 ± 0.7 and 3.8 ± 0.8%; respectively), methyl amyl acetate (2.2 ± 0.4%), viridiflorol (1.2 ± 0.1 and 1.0 ± 0.2%; respectively), rosifoliol (0.6 ± 0.1 and 0.5 ± 0.1%; respectively) and a relatively high content of *p*-cymene (11.6 ± 0.2 and 18.4 ± 0.5%; respectively), *α*-pinene (21.3 ± 0.9 and 13.4 ± 3.8%; respectively) and an average percentage of aromadendrene (2.3 ± 0.2 and 2.3 ± 0.1%; respectively) and *1,8*-cineole (28.7 ± 0.2 and 31.1 ± 5.4%; respectively) (Table 1). This activity was significantly decreased with the mp combination (9.3 ± 2.3 mm; izd), which differed from paac and pwac blend by a higher content of 1,8-cineole (42.8 ± 3.4%), *trans*-pinocarveol (4.3 ± 0.9%), *α*-terpineol (1.1 ± 0.1%) and *β*-pinene (3.1 ± 1.4%) and by a lower level of globulol (0.6 ± 0.2%), viridiflorol (0.2 ± 0.0%), methyl amyl acetate (tr), *α*-pinene (7.0 ± 1.0%) and *p*-cymene (14.6 ± 0.1%).

Therefore, the combination of these two results allowed us to deduce that the increased activity against *H. influenzae* could be attributed to a synergistic effect between globulol, viridiflorol, methyl amyl acetate, *α*-pinene, and *p*-cymene as well as the average percentage of *1,8*-cineole. However, the antagonistic effect could be attributed to the dominance of *1,8*-cineole, which, by interacting with other compounds such as *trans*-pinocarveol, *α*-terpineol, and *β*-pinene may enhance the antibacterial resistance. These observations were supported by several studies, which reported that *Eucalyptus* oils rich in 1,8-cineole and other major components exhibited weak antimicrobial activity; however, the presence of a medium content of 1,8-cineole, with other terpenes such as globulol, *α*-pinene, and p-cymene in *Eucalyptus* oils could produce a synergistic or additive effect [22, 23]. It was also reported that the hyrodrphobic terpenes, *α*-pinene, *p*-cymene had a low water solubility and low hydrogen-bonding capacity, which resulted in a lack of their antibacterial activity [24, 25]. However, these compounds could be accumulated in cell membranes, disturbing their integrity as well as increasing their permeability [18]. Therefore, it could facilitate the transfer of the active components, such as the terpenoids, and enhance the antimicrobial activity.

#### H. parainfluenzae

The most effective activity against this bacterial strain was observed with pwac and bpac EO blends (24.0 ± 5.3 and 23.0 ± 1.7 mm; izd; respectively). These blends were a chemotype of *1,8*-cineole (31.1 ± 5.4 and 34.8 ± 2.0%; resp.), *α*-pinene (13.4 ± 3.8 and 14.4 ± 5.1%; resp.), *p*-cymene (18.4 ± 0.5 and 10.5 ± 0.4%; resp.), aromadendrene (2.3 ± 0.1 and 4.1 ± 0.8%; reszp), globulol (3.8 ± 0.8 and 4.4 ± 0.1%; resp.), spathulenol (1.0 ± 0.8 and 2.1 ± 2.2%; resp.), and viridiflorol (1.0 ± 0.2 and 1.1 ± 0.2%; resp.). The paac blend, belonging to almost the same chemotype as bpac and pwac blends, differed from them by its significantly higher content in α-pinene (21.3 ± 0.9%), showing lower antibacterial activity against the same strain (10.3 ± 0.6 mm; izd). We suggest that a high level of the monoterpene hydrocarbons (*α*-pinene) could interact with the oxygenated terpenes and produce an antagonistic effect against this strain. Several studies reported that the antimicrobial activity of EO blends was not attributed essentially to the main active compounds but also to the interaction between different components present in the EOs, which can have synergistic or antagonistic effects. It also depends on the yield content, concentration, and susceptibility of the tested microorganisms [8, 26].

Therefore, the inactive compounds might influence resorption or the kinetics of the reactions, modulating the biological activities of the tested compounds. It is worth noting that the combination of both major and minor components can modify the biological activity of the EOs and exert a significant synergistic or antagonistic effect [27, 28].

#### Pseudomonas aeruginosa

The most effective activity against this bacterial strain was observed with ac and bp blends (14.3 ± 0.6 and 15.0 ± 0.0 mm; izd; respectively), belonging to different chemotypes but sharing a relatively high average percentage of aromadendrene (2.6 ± 2.8 and 3.7 ± 1.9%; respectively), globulol (7.6 ± 1.6 and 1.2 ± 0.4%; respectively), and a medium content of 1,8-cineole (33.6 ± 5.5 and 36.0 ± 1.5%; respectively). However, this activity was significantly lower than that observed with other blends, particularly against *H. influenzae*. The antagonistic effect against *P. aeruginosa* was observed with paac, pwac, pw, and pwpm blends. These blends are characterized by moderate mean percentages of *1,8*-cineole (28.7 ± 0.2%–35.7 ± 4.3%), but differ from other oil blends by their relatively high contents of *p*-cymene (11.6 ± 0.2%-32.3 ± 2.5%), *γ*-terpinene (0.1 ± 0.0%-2.0 ± 0.9%), and cryptone (2.1 ± 0.5%-4.1 ± 1.0%). In fact, this effect was also observed with EO blends rich in methyl amyl acetate, such as pwac and bpac blends (6.0 ± 0.0 mm; izd).

Comparative analysis of the discussed above results suggests that the synergistic effect observed against *P. aeruginosa* may be mainly due to the interaction between *1,8*-cineole, aromadendrene, and globulol; whereas the presence of a relatively high mean percentage of *p*-cymene, *γ-*terpinene, cryptone, and methyl amyl acetate could significantly reduce antibacterial activity.

It was reported that the *E. gobulus* oil fruit rich in *1,8*-cineole, globulol, and aromadendrene did not show a substantial inhibition against the Gram negative bacterial strains *P. aeruginosa*, *K. pneumoniae*, and *E. coli*. However, combinations of aromadendrene and *1,8*-cineole could show an additive or synergistic effects against Gram positive bacteria, when the dose of *1,8*-cineole is significantly higher than that of aromadendrene [29].

#### Escherichia coli

The analysis of the antibacterial activity revealed an antagonistic effect of all tested oil blends against this bacterial strain. For instance, the combination of *E. robusta* oil (r), known by its relatively high content in citronellal,* α*-terpineol, *endo*-borneol, and rosifoliol and a high activity against *E. coli*, with *E. accedens* oil (a), known by its lower content of above components and a significant higher mean percentage of *α*-pinene and almost an equal amount of *1,8*-cineole, showed a significant reduction of the antibacterial activity of their mixture (ra blend); showing antagonistic effect.

The most probable explanation for this antagonism is that a high concentration of the monoterpene hydrocarbon *α*-pinene, reduces aqueous terpene solubility, affecting the antimicrobial activity of the active components such as alcohols *α*-terpineol, *endo*-borneol, and rosifoliol [11, 24, 30, 31]. It was reported previously that the aldehyde component, citronellal, is inactive against *E. coli*, which had also a low water solubility [30], which could affect the solubility of the active components and support this antagonistic effect exerced by the high content of *α*-pinene.

Similarly, the ar blend exhibited an antagonistic effect against *E. coli*, producing a significant reduction of its inhibition growth (8.3 ± 0.6 mm;dzi), comparatively to that produced by their single oils (12,3 ± 3,8 and 20.7 ± 1.5 mm;dzi; resp.). In fact this blend was characterized by a higher mean percentage of the monoterpene hydrocarbons, *α*-pinene (26.7 ± 7.8%), almost the same contents in *1,8*-cineole (27.3 ± 2.9), *p*-cymene (10.2 ± 1.5), and a lower mean percentages in *α*-terpineol, *trans*-pinocarveol, *endo*-borneol, and rosifoliol than those detected in the EO of *E. robusta*, characterized by a strong antibacterial effect against *E. coli*.

All the above discussed results allow us to support the point of view that the antagonistic effect exerced by the hydrophobic mononterpens hydrocarbons *α*-pinene and *p*-cymene could reduce the solubility of the oxygenated compounds and therefore affect their biological activities [32].

#### Klebsiella pneumoniae

As discussed for *E. coli*, the analysis of the antibacterial activity revealed an antagonistic effect of all tested oil blends against this bacterial strain. The wc blend exhibited an antagonistic effect against *K. pneumoniae.* The comparison of the wc EO chemical composition to that of *E. cladocalyx* (c), which showed a strong inhibitory effect against this strain, revealed that the wc blend has almost the same content of *1,8*-cineole, a higher content of *p*-cymene, and a lower mean percentage of globulol, epiglobulol, rosifoliol, viridiflorol, aromadendrene, and methyl amyl acetate.

These results suggest that the decreased antibacterial activity could be explained by the reduction in the mean percentage of active compounds, such as the terpenoids, and by a high concentration of the monoterpene hydrocarbons *p*-cymene. Despite the antagonistic effect shown for the lb blend against the same strain, this activity was significantly increased compared to those produced by the single oils. This difference could be influenced by the difference in the concentration of several compounds involved directly or indirectly in the antibacterial activity. For example, when we compare the chemical composition of the blend lb to that of the single oil of *E. bositoana* (b), which showed a strong activity, we found a small decrease in the concentration of *1,8*-cineole, *α*-pinene, and aromadendrene, an increasing level of terpinen-4-ol, *trans*-pinocarveol, *α*-terpineol, globulol, *β*-pinene, *p*-cymene, and almost the same concentration in spathulenol. We suggest that the increased antibacterial activity could be due to the increasing level of the oxygenated terpenes such as the monoterpene alcohols terpinen-4-ol, trans-pinocarveol, and *α*-terpineol which were linked to their high antibacterial activities against several Gram negative bacteria such as *K. pneumoniae* [33, 34]. Additionally, it was reported that the combination of the two isomers terpinen-4-ol and *α*-terpineol exhibited a synergistic effect on the Gram negative bacteria *Shigella flexneri* [35]. This activity could be supported by the presence of the sesquiterpenic alcohols spathulenol, globulol, as well as by the main compound 1,8-cineole. The later was reported as the principal components responsible in the antibacterial activity against *K. pneumoniae* [36, 37].

However, the monoterpenes hydrocarbons *β*-pinene, *p*-cymene, and *α*-pinene could interact together by reducing the water solubility of the terpenoids, such as the *1,8*-cineole, which known by its low water solubility, and considerably reduces the effect of the active compounds [24]. Therefore, the blend lb showed a slightly increase of its antibacterial activity, compared to that observed with *E. bositoana* oil.

### Antibacterial activity against Gram ( +) bacterial strain

#### Staphylococcus aureus

The analysis showed that the wc and bpac blends, rich in epiglobulol, viridiflorol, globulol, methyl amyl acetate, and aromadendrene, produced an antagonistic effect against this bacterial strain (8.7 ± 2, 3 and 9.0 ± 0.0 mm; izd). However, the mp and wp blends, characterized by their low levels of the above components and almost equal content of *α*-pinene, *β*-pinene, cryptone, and *p*-cymen-8-ol, compared to wc and bpac blends, produced a synergistic effect (24.7 ± 5.0 and 23.30 ± 5.0 mm; izd, respectively).

These findings suggest that compounds such as epiglobulol, viridiflorol, globulol, methyl amyl acetate, aromadendrene, and other minor components may be responsible for the antagonism effect. In fact, these compounds were reported as active against a wide range of bacterial species, including *S. aureus* [24, 29, 38]. However, their combination could have an antagonistic [8].

This mechanism of interaction is not well understood and requires further investigations. It could be explained by an interaction between these compounds to reduce the active terpenes solubility, or by competing for the same binding sites on bacterial active sites [8, 19].

### Correlation of the antibacterial activity of EO blends tested by the broth microdilution method with their chemical compositions

The results showed a synergistic effect of certain binary EO blends, essentially against Gram negative bacterial strains such as *E. coli*, *K. pneumoniae*, *H. influenzae*, and *H parainfluenzae*. However, none of the combined of binary blends has shown synergistic. These results were in concordance with those reported by Kachkoul et al. [39].

The active binary blends (pw, pm, pa, bp, and bl), were composed of single EOs with low antibacterial activity, such as *E. punctata* (p) and a medium or high activity, such as *E. wandoo* (w), *E. melliodora* (m), and *E. accedens* (a). These blends, except bl blend, are belonging to the same chemical group according to the ACP analysis (group IIc) and sharing together the highest mean percentage in monterpenic hydrocarbons, monoterpenic alchols, monoterpenic aldehydes, and ketones. All of these chemicals are represented essentially by p-cymene, *trans*-pinocarveol, cuminal, and cryptone, respectively. They are characterized by a medium content in *1,8*-cineole. However, bl, bm, and blbm blends, belonging to another chemical group, characterized by their high content in *1,8*-cineole, terpine-4-ol and lower content in *p*-cymene and cryptone, cuminal and *p*-cymen-8-ol, showed an antagonistic effect against *E. coli*, *K. pneumoniae*, *H. influenzae*, *P. aeruginosa*, and *H. parainfluenzae*.

The results suggest that the presence of a relatively low to medium content in *1,8*-cineole and a medium to high mean percentage in *p*-cymene, as well as the presence of cryptone, cuminal, and *p*-cymen-8-ol produced a synergistic effect against these strains. It was reported that terpenic components can interact to either decrease or increase antimicrobial activity, depending upon their relative concentrations and the overall susceptibility of the target microorganism. Moreover hydrophobic terpenes such as *p*-cymene and *α*-pinene are expected to be accumulated preferentially in the cell membranes to facilitate the absorption of the active compounds such as *1,8*-cineole, shown to be active against *E. coli* [26, 40]. Another study reported that hydrophilic EO components such as cryptone and *p*-cymen-8-ol showed a high hydrogen binding, promoting the entrance of hydrophilic components; whereas hydrocarbon terpenes, such as limonene, provide higher enhancing activity for the lipophilic components [41].

### Comparison between disc diffusion, broth microdilution methods and their activities against the bacterial strains

We highlight in the present study a discordance in the antibacterial activity results obtained by the disc diffusion and the broth microdilution methods. This observed discordance could be related to several factors: i) the low diffusion ability of some active chemical compounds through the agar, which in itself is highly dependent on their polarity and on water solubility; ii) the chemical structure of the EOs’ components and their molecular properties; iii) their site and mode of action, and their interaction with the surrounding environment [19, 24].

Some differences in the obtained results of the present study could be attributed to the tested bacterial strains and the chemical compositions of EO blends.

In our study, three binary EO blends (pm; pw, and ac) showed higher antibacterial activity against Gram-positive bacteria (*S. aureus*) using disc diffusion method than against Gram-negative bacteria. However, the later was more sensitive to five EO blends (bl, bp, pa, pm, and pw) using broth microdilution method (Table 5.supl).

For example the blends pw and pm, which differed significantly in their content in *1,8*-cineole and sharing almost the same mean percentage in monoterpene alcohols *trans*-pinocarveol, *α*-terpineneol, terpinen-4-ol, *p*-ymen-8-ol, had a synergistic effect against *S. aureus* by the disc diffusion method, however they showed an antagonistic effect by the broth microdilution method. This difference in antibacterial effects was also observed with ac blend, characterized by its high mean percentage of the sesquiterpene alcohols globulol, epiglobulol, viridiflorol, and the ester methyl amyl acetate.

We suggest that the activity could be due to the presence of monoterpene and sesquierpenes alcohols and their interactions, as they are known by their high water solubility and high affinity to cell membrane of bacterial strains [42].

Additionally, the Gram(-) bacteria is constituted by a lipopolysaccharide layer (LPS) which acts as a barrier for macromolecules and hydrophobic compounds, such as those present in the above discussed chemical components [43, 44]. Therefore, the Gram (-) bacteria are more resistance against the hydrophobic active components than the Gram-positive bacteria. Indeed, the hydrophobicity properties of EO components and the synergistic action of their components enable them to accumulate in cell membranes, which can change the fluidity, disturb the structures and cause the increase of the cell membrane penetrability [45].

In another hand, the Gram( +) bacteria sensitivity to some EO components could be attributed to the membrane structure, as they lack the LPS layer [46].

## Conclusion

To the best of our knowledge, this study marks a pioneering demonstration of the antibacterial properties of *Eucalyptus* sp. EOs combinations against the microorganisms responsible for ear infections. Our investigation uncovered an enhancement in the efficacy of these EOs when combined at specific proportions, albeit not all potential combinations were explored.

The effectiveness of these essential oil blends exhibited variation depending on the targeted microorganism and the diverse chemical constituents within the mixture. These versatile combinations present an alternative to conventional medications, which are progressively becoming less effective against numerous microorganisms responsible for significant ear infections. The broad array of chemical ingredients and potential synergistic effects among compounds make these combinations promising in the treatment of such infections.

### Supplementary Information

Supplementary Material 1.

## Data Availability

Data and materials are available from authors on reasonable request.

## Abbreviations

GC: Gaz chromatography
GC/MS: Gaz chromatography coupled to the mass spectroscopy
HP: Hewlett-Packard
FID: Flame ionization detector
cap: Capillary
anal: Analytical
i.d.: Internal diamaeter
EOs: Essential oils
MH: Mueller–Hinton
MIC: Minimal inhibition concentration
MBC: Minimum bactericidal concentration
IZD: Inhibition zone diameter
DMSO: Dimethyl sulfoxide
PCA: Principal components analysis
HCA: Hierarchical clusters analysis
RI: Retention index

## References

[1] Hailu D, Mekonnen D, Derbie A, Mulu W, Abera B (2023). Pathogenic bacteria profile and antimicrobial susceptibility patterns of ear infection at Bahir Dar Regional Health Research Laboratory Center. Ethiopia SpringerPlus.

[2] Worku M, Bekele M (2014). Bacterial isolate and antibacterial resistance pattern of ear infection among patients attending at Hawassa university referral Hospital, Hawassa. Ethiopia Indian J Otol.

[3] Argaw-Denboba A, Abejew AA, Mekonnen AG (2016). Antibiotic-resistant bacteria are major threats of otitis media in Wollo area, Northeastern Ethiopia: a ten-year retrospective analysis. Int J Microbiol.

[4] Ghaly MF, Shaheen AA, Bouhy AM, Bendary MM. Alternative therapy to manage otitis media caused by multidrug-resistant fungi. Arch Microbiol J 2020. 10.1007/s00203-020-01832-z. 10.1007/s00203-020-01832-z 32108246

[5] Panahi Y, Akhavan A, Sahebkar A, Hosseini SM, Taghizadeh M, Akbari H (2014). Investigation of the effectiveness of Syzygium aromaticum, Lavandula angustifolia and Geranium robertianum essential oils in the treatment of acute external otitis: a comparative trial with ciprofloxacin. J Microbiol Immunol Infect.

[6] Becerril R, Nerín C, Gómez-Lus R. Evaluation of bacterial resistance to essential oils and antibiotics after exposure to oregano and cinnamon essential oils. 201210.1089/fpd.2011.1097 10.1089/fpd.2011.1097 22827568

[7] Yap PSX, Yiap BC, Ping HC, Lim SHE (2014). Essential oils, a new horizon in combating bacterial antibiotic resistance. Open Microbiol J.

[8] Bassolé IHN, Juliani HR (2012). Essential oils in combination and their antimicrobial properties. Molecules.

[9] Samoussa MO, Abdellaoui A, Kettani A, Saile R; Bennani H. Étude de la Sensibilité Aux Huiles Essentielles de Cinnamomum Verum, Eucalyptus Globulus, et Glycyrrhiza Glabra L Ainsi qu’aux Antibiotiques de Certains Germes Issus de la Restauration Collective. Eur Sci J. 2018. 10.19044/esj.2018.v14n3p584.

[10] Ayari S, Shankar S, Follett P, Hossain F, Lacroix M. Potential synergistic antimicrobial efficiency of binary combinations of essential oils against Bacillus cereus and Paenibacillus amylolyticus-Part A. Microb Pathog. 2022. 10.1016/j.micpath.2020.104008. 10.1016/j.micpath.2020.104008 31991163

[11] Ameur E, Sarra M, Yosra D, Mariem K, Nabil A, Lynen F, Larbi KM. Chemical composition of essential oils of eight Tunisian Eucalyptus species and their antibacterial activity against strains responsible for otitis. BMC Complement Med Ther. 2021. 10.1186/s12906-021-03379-y. 10.1186/s12906-021-03379-y PMC8359536 34384412

[12] Fadil M, Fikri-Benbrahim K, Rachiq S, Ihssane B, Lebrazi S, Chraibi M, et al. Combined treatment of Thymus vulgaris L., Rosmarinus officinalis L. and Myrtus communis L. essential oils against Salmonella typhimurium: Optimization of antibacterial activity by mixture design methodology. Eur J Pharm Biopharm. 2018. 10.1016/j.ejpb.2017.06.002. 10.1016/j.ejpb.2017.06.002 28583590

[13] Schaechter M, Medoff G, Eisenstein BI, Flandrois J-P (1999). Microbiologie et pathologie infectieuse (Français) Broché –.

[14] NCCLS. Performance Standards for Antimicrobial Disk and Dilution Susceptibility Tests for Bacteria Isolated from Animals, Approved Standard. 2nd Edition, NCCLS Document M31-A2 22(6). Wayne: Clinical and Laboratory Standards Institute; 2002.

[15] Eugénie BB, Brogardugénie EJM (1999). Bases biologiques de l’antibiothérapie.

[16] Sovcíková A, Mikulásová M, Horáková K, Floch L (2001). Antibacterial and mutagenic activities of new isothiocyanate derivatives. Folia Microbiol (Praha).

[17] Bhat AS, Ahangar AA. Methods for detecting chemical–chemical interaction in toxicology. Toxicol Mech and Methods. 2007. 10.1080/15376510601177654. 10.1080/15376510601177654 20020870

[18] Lv F, Liang H, Yuan Q, Li C. In vitro antimicrobial effects and mechanism of action of selected plant essential oil combinations against four foodrelated microorganisms. Food Res Int. 2011. 10.1016/j.foodres.2011.07.030

[19] Hyldgaard M, Mygind T, Meyer RL. Essential oils in food preservation: mode of action, synergies, and interactions with food matrix components. Front Microbiol. 2012. 10.3389/fmicb.2012.00012. 10.3389/fmicb.2012.00012 PMC3265747 22291693

[20] Nikbakht MR, Rahimi-Nasrabadi M, Ahmadi F, Gandomi H, Abbaszadeh S, Batooli H. The chemical composition and in vitro antifungal activities of essential oils of five eucalyptus species. Essent. Oil-Bear. Plants. 2015. 10.1080/0972060X.2014.935061.

[21] Ray S, Jena S, Dash B, Kar B, Halder T, Chatterjee T et al. Chemical diversity, antioxidant and antimicrobial activities of the essential oils from Indian populations of Hedychium coronarium Koen. Ind Crops Prod. 2018. 10.1016/j.indcrop.2017.12.033.

[22] Hendry ER, Worthington T, Conway BR, Lambert PA. Antimicrobial efficacy of eucalyptus oil and 1,8-cineole alone and in combination with chlorhexidine digluconate against microorganisms grown in planktonic and biofilm cultures. J Antimicrob Chemother. 2009. 10.1093/jac/dkp362. 10.1093/jac/dkp362 19837714

[23] Bakkali F, Averbeck S, Averbeck D, Idaomar M. Biological effects of essential oils – A review. Food Chem Toxicol. 2008. 10.1016/j.fct.2007.09.106. 10.1016/j.fct.2007.09.106 17996351

[24] Griffin SG, Wyllie SG, Markham JL, Leach DN. The role of structure and molecular properties of terpenoids in determining their antimicrobial activity. Flavour Fragr J. 1999. 10.1002/(SICI)1099-1026(199909/10)14:5%3c322::AID-FFJ837%3e3.0.CO;2-4.

[25] Nazzaro F, Fratianni F, De Martino L, Coppola R, De Feo V. Effect of Essential Oils on Pathogenic Bacteria. Pharmaceuticals (Basel). 2013. 10.3390/ph6121451. 10.3390/ph6121451 PMC3873673 24287491

[26] Gallucci MN, Oliva M, Casero C, Dambolena J, Luna A, Zygadlo J, et al. Antimicrobial combined action of terpenes against the food-borne microorganisms Escherichia coli, Staphylococcus aureus and Bacillus cereus. Flavour Fragr J. 2009. 10.1002/ffj.1948.

[27] Semeniuc CA, Pop CR, Rotar AM. Antibacterial activity and interactions of plant essential oil combinations against Gram-positive and Gramnegative bacteria. J Food Drug Anal. 2017. 10.1016/j.jfda.2016.06.002. 10.1016/j.jfda.2016.06.002 PMC9332530 28911683

[28] Stephane FFY, Jules BKJ. Terpenoids as Important Bioactive Constituents of Essential Oils. Essential Oils - Bioactive Compounds, New Perspectives and Applications. IntechOpen. 2020. 10.5772/intechopen.91426.

[29] Mulyaningsih S, Sporer F, Zimmermann S, Reichling J, Wink M. Synergistic properties of the terpenoids aromadendrene and 1,8-cineole from the essential oil of Eucalyptus globulus against antibiotic-susceptible and antibiotic-resistant pathogens. Phytomed. 2010. 10.1016/j.phymed.2010.06.018. 10.1016/j.phymed.2010.06.018 20727725

[30] Cox SD, Mann CM, Markham JL. Interactions between components of the essential oil of Melaleuca alternifolia. J Appl Microbiol. 2001. 10.1046/j.1365-2672.2001.01406.x. 10.1046/j.1365-2672.2001.01406.x 11556915

[31] Li L, Shi C, Yin Z, Jia R, Peng L, Kang S, et al. Antibacterial activity of α-terpineol may induce morphostructural alterations in Escherichia coli. Braz J Microbiol. 2015. 10.1590/s1517-83822014000400035. 10.1590/s1517-83822014000400035 PMC4323317 25763048

[32] Fandiño I, Fernandez-Turren G, Ferret A, Moya D, Castillejos L, Calsamiglia S. Exploring Additive, Synergistic or Antagonistic Effects of Natural Plant Extracts on In Vitro Beef Feedlot-Type Rumen Microbial Fermentation Conditions. Animals: an Open Access Journal from MDPI [Internet]. Disponible sur: 2020;10(1). [cité 13 mars 2023]. https://www.ncbi.nlm.nih.gov/pmc/articles/PMC7022539/. 10.3390/ani10010173 PMC7022539 31968596

[33] Cheruvanachari P, Pattnaik S, Mishra M, Pragyandipta P, Naik PK. Terpinen4-ol, An Active Constituent of Kewda Essential Oil, Mitigates Biofilm Forming Ability of Multidrug Resistant Staphylococcus aureus and Klebsiella pneumoniae. J Biol Act Prod. 2022. 10.1080/22311866.2022.2154264.

[34] Johansen B, Duval RE, Sergere JC. First Evidence of a Combination of Terpinen-4-ol and α-Terpineol as a Promising Tool against ESKAPE Pathogens. Molecules. 2022. 10.3390/molecules27217472. 10.3390/molecules27217472 PMC9654757 36364298

[35] Huang J, Yang L, Zou Y, Luo S, Wang X, Liang Y, et al. Antibacterial activity and mechanism of three isomeric terpineols of Cinnamomum longepaniculatum leaf oil. Folia Microbiol. 2021. 10.1007/s12223-020-00818-0. 10.1007/s12223-020-00818-0 32895862

[36] Shahverdi A. Enhancement of antimicrobial activity of furazolidone and nitrofurantoin against clinical isolates of Enterobacteriaceae by piperitone. Int J Aromather. 2004. 10.1016/j.ijat.2004.04.00.

[37] Winnett V, Sirdaarta J, White A, Clarke FM, Cock IE. Inhibition of Klebsiella pneumoniae growth by selected Australian plants: natural approaches for the prevention and management of ankylosing spondylitis. Inflammopharmacol. 2017. 10.1007/s10787-017-0328-1. 10.1007/s10787-017-0328-1 28239782

[38] Mulyaningsih S, Sporer F, Reichling J, Wink M. Antibacterial activity of essential oils from Eucalyptus and of selected components against multidrug-resistant bacterial pathogens. Pharm Biol. 2011. 10.3109/13880209.2011.553625. 10.3109/13880209.2011.553625 21591991

[39] Kachkoul R, Touimi GB, Bennani B, Habbani RE, Mouhri GE, Mohim M, et al. The synergistic effect of three essential oils against bacteria responsible for the development of lithiasis infection: an optimization by the mixture design. eCAM. 2021. 10.3390/ani10010173. 10.1155/2021/1305264 PMC8421168 34497653

[40] Zengin H, Baysal A. Antibacterial and antioxidant activity of essential oil terpenes against pathogenic and spoilage-forming bacteria and cell structure-activity relationships evaluated by SEM microscopy. Molecules. 2014. 10.3390/molecules191117773. 10.3390/molecules191117773 PMC6272013 25372394

[41] El-Kattan AF, Asbill CS, Kim N, Michniak BB. The effects of terpene enhancers on the percutaneous permeation of drugs with different lipophilicities. Int J Pharm. 2001. 10.1016/s0378-5173(00)00699-2. 10.1016/s0378-5173(00)00699-2 11250108

[42] He Y, Sang S, Tang H, Ou C. In vitro mechanism of antibacterial activity of eucalyptus essential oil against specific spoilage organisms in aquatic products. J Food Process Preserv. 2022. 10.1111/jfpp.16349.

[43] Ouattara B, Simard RE, Holley RA, Piette GJP, Bégin A. Antibacterial activity of selected fatty acids and essential oils against six meat spoilage organisms. Int J Food Microbiol. 1997. 10.1016/s0168-1605(97)00070-6. 10.1016/s0168-1605(97)00070-6 9310850

[44] Pandey AK, Kumar P, Singh P, Tripathi NN, Bajpai VK. Essential Oils: Sources of Antimicrobials and Food Preservatives. Front Microbiol. 2017. [cité 20 mars 2023]. 10.3389/fmicb.2016.02161. 10.3389/fmicb.2016.02161 PMC5238431 28138324

[45] Bajpai VK, Sharma A, Baek KH. Antibacterial mode of action of Ginkgo biloba leaf essential oil: Effect on morphology and membrane permeability. Bangladesh J Pharmacol. 2015. 10.3329/bjp.v10i2.22546.

[46] Gilles M, Zhao J, An M, Agboola S. Chemical composition and antimicrobial properties of essential oils of three Australian Eucalyptus species. Food Chem. 2010. [cité 13 oct 2021]. 10.1016/j.foodchem.2009.07.021.

