# Supplementary material for: Chemical compositions of Eucalyptus sp. Essential oils and the evaluation of their combinations as a promising treatment against ear bacterial infections
Source: BMC Complement Med Ther. 2024 Jun 7;24:220. doi: 10.1186/s12906-024-04494-2 (PMC11157906; doi:10.1186/s12906-024-04494-2)
Supplement: Supplementary file 1 — Supplementary Material 1. [file 12906_2024_4494_MOESM1_ESM.docx]

Table 1. Supl. Effect of EOs blends according to the bacterial strains and to the used method

| **Eos blends** | Bactrial strains | Disc method | Broth microdilution method |
| --- | --- | --- | --- |
| pa | *E. coli* | - | + |
| pw |  |  |  |
| bp |  |  |  |
| pw | *H. parainfluenaze* | - | + |
| pw | *K. pneumoniae* | - | + |
| bl |  |  |  |
| pa |  |  |  |
| pm |  |  |  |
| ac | *S. aureus* | + | - |
| pm |  |  |  |
| pw |  |  |  |
| bm | *H influenzae* | + | - |
| pa |  |  |  |
| bp | *P. aeruginosa* | + | - |
| ac |  |  |  |
| bmpm | *H. influenzae*  *H. influenzae* | + | - |
| pwac |  |  |  |
| pwpm |  |  |  |
| bpac | *H. parainfluenzae*  *H. parainfluenzae* | + | - |
| pwac |  |  |  |
| pwpm |  |  |  |

|  |
| --- |
